# Evaluating mobile services using integrated weighting approach and fuzzy VIKOR

**DOI:** 10.1371/journal.pone.0217786

**Published:** 2019-06-04

**Authors:** Yongyoon Suh, Yongtae Park, Daekook Kang

**Affiliations:** 1 Department of Safety Engineering, Pukyong National University, Busan, Republic of Korea; 2 Department of Industrial Engineering, Seoul National University, Seoul, Republic of Korea; 3 Department of Industrial and Management Engineering, Inje University, Gimhae-si, Gyeongsangnam-do, Republic of Korea; Shandong University of Science and Technology, CHINA

## Abstract

Mobile services’ rapid evolution and development has meant that their evaluation has become a more and more pressing issue, and from both the practical and theoretical standpoints. The significant previous work in the field of multiple-criteria decision-making based evaluation of mobile services has some practical limitations that should be noted. First, there has been insufficient research that has utilized both objective and subjective weighting. Second, the investigations that have employed Vise Kriterijumska Optimizacija I Kompromisno Resenje (VIKOR), a well known practical tool for use in multi-criteria decision making, did not consider the fuzzy environment. In order to fill these gaps in the literature, the present study developed fuzzy VIKOR for use with an integrated weighting approach that combines subjective and objective weighting to account for mobile services’ various characteristics and, thereby, evaluate their quality. For subjective weighting, Decision Making Trial and Evaluation Laboratory (DEMATEL) was employed for simple determination of the weighting and causal relationships. For objective weighting of evaluation criteria, Shannon entropy was utilized. This study has a unique contribution in that it reflects the special circumstances of the mobile service evaluation that have not been considered in the previous studies. Especially, in this study, not only the subjective weighting method but also the objective weighting method are used for more accurate importance weight of evaluation criteria. In the novelty aspect, this is the first study trying to utilize fuzzy VIKOR in concert with a novel combined subjective/objective weighting method in order to integrate objective decision-matrix-derived information with subjective decision-maker preferences. Additionally, a supplemental, empirical mobile-service-evaluation case study was conducted that enables researchers and practitioners to better understand the overall, practical evaluation process. Validation of the case study results by comparison with other, representative multiple-criteria decision-making methods verified the proposed method’s robustness.

## Introduction

Advances in wireless communication technology have increased the use of mobile devices and also accelerated the development of mobile services [1]. Especially, mobile service business has entered a new era with the advent of mobile devices with new features and the evolution of the mobile app ecosystem since the Apple App Store launched on July 10, 2008. [2, 3]. Besides, the range and scope of mobile services has dramatically expanded from traditional service such as SMS and e-mail to wide range of businesses and services such as streaming video, navigation, social networking, and location-based services. Under this circumstance, companies have strived for competitive edges in the mobile service marketplace [2]. The surprisingly rapid emergence and of this new service paradigm based on information and communication technology has raised core issues related with the customer’s continued satisfaction with mobile services [4]. Thus, evaluation of customer satisfaction in mobile service has become greatly important [5]. Mobile service evaluation is a very high priority for both service providers and customers [6]. For service providers, information on customers’ recognitions of service levels can be very valuable data that can be utilized to improve their service quality. For customers meanwhile, it is helpful to be able to weigh the advantages and disadvantages of each mobile service, each offering similar and comparable functionalities, before making any purchase decision. Academic research involving mobile service evaluation, however, is scarce. Hence, the issue of mobile service evaluation has become more significant.

Many researchers have recognized the mobile service evaluation as an issue of multiple-criteria decision-making (MCDM) and applied various MCDM methods to date. An overview of the commonly used MCDM methods for the different problem applied by the previous researchers is revealed briefly in Table 1.

**Table 1:** Application of MCDM methods for the different problem.

Type	Method	Author(s)	Problem
Applying applied analytic hierarchy process (AHP) or analytic network process (ANP)	fuzzy analytic hierarchy process (FAHP)	Büyüközkan [7]	Determining the mobile commerce user requirements
	AHP	Nikou and Mezei [8]	Identifying the most preferred service category based on users’ preferences and the most influencing factors for mobile service adoption
	FAHP	Shieh, Chang [9]	Analyzing the key attributes that affect the mobile service adoption in Taiwan
	ANP	Chen and Cheng [10]	Finding the best strategy of mobile service providers for delivering mobile services
Utilizing a hybrid MCDM approach	DEMATEL and ANP	Jyh-Fu Jeng and Bailey [11]	Investigating the customer retention factors in the mobile telecom sector
	DEMATEL, ANP and VIKOR	Lu, Tzeng [12]	Exploring the effect of user behavior and guidance on the mobile banking services
	VIKOR and sentiment analysis	Kang and Park [6]	Evaluating customer satisfaction level in mobile service using customer review data

Although all the aforementioned studies enlighten the evaluation of mobile service, there are two important issues that have not been addressed in depth in previous studies.

1. *How can we accurately measure the importance weight*?
2. *Among the various MCDM methods*, *what is the most appropriate MCDM method for evaluating mobile services*?

Regarding the *first* issue, in the literature, scholars have begun to study how to effectively weight criteria to make decision-making more scientific. The approaches for weighting criteria can be divided into two categories: subjective weighting and objective weighting [13, 14]. The former, such as AHP and DEMATEL, collects the subjective preferences of the decision makers. Subjective weighting method can accurately reflect all decision makers’ different opinions on criteria weights because the weights of criteria are determined solely based on the preferences or judgments of decision makers. In previous studies, various subjective weighting methods such as AHP [7–9], ANP [10, 15], DEMATEL with ANP [16, 17] were utilized for evaluation of mobile service. On the other hand, objective weighting method is based on data that are given in the decision matrix of the attributes for each alternative. This method uses entropy or multiple objective programming to exclude subjective preferences from decision makers. [14, 18]. In a particular evaluation environment, the evaluator's subjectivity can often bias the evaluation results. Thus, in this case, the objective weighing method can be utilized. This objective weighting method can overcome the shortcomings of the subjective approach by eliminating man-made instabilities and yielding more realistic results [19]. This method has been applied for various decision making problems including location selection [20], renewable energy source evaluation [21], reverse logistics problem[22] supplier selection [14], industrial robot selection [23] and service quality evaluation such as airline service [24]. However, there is lack of research applying objective weighting method for evaluating mobile service despite the need. In the mobile service evaluation, because there are many evaluation criteria causing the uncertainty [25], both subjective preferences of experts and objective assessment information are important. Thus, because the objective weighting method can be used effectively in uncertain environments where subjective weight cannot be applied [26], combining objective weighting with subjective weighting can be rational approach for calculating weights of criteria for mobile service evaluation.

Regarding the *Second* issue, in previous studies, there are many ways to measure quality of service such as QFD (Quality Function Deployment), PROMETHEE (Preference Ranking Organization Method for Enrichment Evaluation), and TOPSIS (Technique for Order Preference by Similarity to Ideal Solution, VIKOR, and AHP. Amongst these methodologies, VIKOR has been well utilized for evaluating mobile service [17, 27], because it is recognized as a practical tool in multi-criteria decision making, especially when the decision makers are not aware of how to express preference in the decision making processes [28]. Compared with the other MCDM methods, VIKOR has some advantages for evaluating mobile service. When a customer selects one of their preferred mobile services, they exclude other mobile services with unsatisfactory characteristics in the initial screening step [6]. In fact, this step is very important for mobile service selection. In other words, when choosing a mobile service, customers consider not only the total score for all criteria, but also the score for the most unsatisfactory criteria. For this situation, VIKOR is advantageous over other MCDM methods because it can derive the ranking order taking into consideration both a maximum ‘‘group utility of the majority”, which is a utility reflecting the consideration of all relevant criteria, and the minimum ‘‘individual regret of the opponent”, which is the most unsatisfactory criteria considerations. This approach not only becomes a compromise foundation ground for mutual communication, negotiation and conflict management, but also acts as a bridge between decision makers. In addition, multi-dimensional consideration of higher and lower performance ratings of feasible alternatives can help decision makers keep away from making inappropriate decisions [29].

However, previous studies utilizing VIKOR as evaluation method for mobile service does not consider fuzzy environment. Mobile service quality perceptions depend on the linguistic aspects and preferences of different decision makers, so this issue should be conducted in an uncertain and fuzzy environment. In recent years, fuzzy-based methodologies have been used to address uncertain MCDM problems. These fuzzy-based methodologies contain defuzzification process that transforms linguistic variables such as “very good” or “poor” intro crisp values for more scientific and precise evaluation. Because it is the advanced approach, previous studies utilizing VIKOR for mobile service evaluation did not consider defuzzification process. To solve the problems that the information on attributes or alternatives is uncertain and inconsistent, we use fuzzy sets theory and the VIKOR to evaluate the mobile service quality because it presents a compromise resolution in an ambiguous, unclear and uncertain environment.

Motivated by first and second issues raised by previous studies, this study presents a new MCDM approach by utilizing integrated weighting approach and fuzzy VIKOR for evaluation of mobile service quality. To solve the *first* issue, we utilize the integrated weighting approach. By utilizing both subjective and objective weight, we can monitor how the evaluation results vary according to the weighting approaches. Regarding with subjective weighting method, DEMATEL is utilized in this study because it can calculate importance coefficient by reflecting causal relationships among criteria without complicated process. These questions may arise. "Why is it better to use DEMATEL instead of other methodologies as a subjective weighting method?". In the case of AHP or ANP, which is a representative subjectvie weighting method, it is advantageous to calculate the weight effectively through pairwise comparison, but a lot of additional information is needed. However, in the evironment of mobile service evaluation, it is not easy for the evaluator to perform pair comparison for all evaluation criteria due to lack of information. Compared with AHP and ANP, DEMATEL does not cause the complexity in the evaluation process because additional processes such as obtaining the weighted supermatrix and limiting the weighted supermatrix should be needed when using ANP method. In other words, compared with AHP or ANP, it is advantageous to calculate the weight effectively even with a smaller amount of information. In addition, in mobile service, a dynamic interrelationship between criteria should be taken into consideration. Because mobile services are delivered through mobile devices, mobile service evaluation should be considered *service-based criteria* [30–33] and *product-based criteria* [34–37]. *Service-based criteria*, which are criteria related to services themselves, include a service level considering mobile technology advancement and the diversity of its content provision; *product-based criteria* are related to hardware performance of mobile device, which can affect mobile service quality indirectly such as LCD resolution. These *service-based* and *product-based criteria*, unavoidably, are interrelated. For instance, there are a conflict between GPS service accuracy, which is a *service-based criterion*, and device portability, which is a p*roduct-based criterion*, are in conflict, because for example, improving GPS accuracy generally increases the size and weight of mobile devices, in other words, it reduces the portability of mobile devices. Thus, it is needed to consider both *service-based* and *product-based criteria* and their interrelationship. The DEMATEL methodology, which has the advantage of capturing dynamic interrelationship between criteria, can be a good alternative. In DEMATEL, based on the derived interrelationship between criteria using influence relation map, importance weights can be calculated systematically. In terms of objective weighting method, Shannon entropy, well-known as an objective approach to identifying weights of evaluation criteria, is appropriate for measuring the relative contrast intensity of an attribute to indicate the average internal information sent to a decision maker [13]. It does not need to collect the subjective perceptions of decision makers, but collect the performance of the evaluation objects, e.g. criteria [38]. For these reasons, this study utilizes DEMATEL and Shannon entropy as criteria weighting method. The advantage of the integrated weighting approach utilized in this study is that the rate of reflection for two types of weighting approaches can be adjusted based on decision makers’ own responsibilities considering the different characteristics of evaluation system. In other words, in previous studies, only one of subjective weight or objective weight was included in the evaluation, so sensitivity analysis could not be performed. However, in this study, the sensitivity analysis can be performed by adjusting the weighting of the subjective weight and the objective weight in consideration of the inherent characteristics of the mobile service and the evaluation environment.

To solve the *second* issue, this study uses the fuzzy VIKOR. This fuzzy VIKOR method can handle the uncertainties in the mobile service evaluation effectively. In addition, the VIKOR can evaluate mobile service alternatives and analyze gaps in the desired level of performance for each mobile service systematically.

This study has a unique contribution in that it reflects the special circumstances of the mobile service evaluation that have not been considered in the previous studies. Specifically, in the previous research, subjective weighting methods were applied mainly to derive the weight of mobile service evaluation criteria. However, in this study, not only the subjective weighting method but also the objective weighting method are used for more accurate importance weight of evaluation criteria. In other words, this study endeavored to fill the gaps in the literature by suggesting a new integrated weighting approach that has never been applied to mobile service evaluation. In addition, there have been no studies combining the fuzzy theory and the VIKOR method for evaluating mobile services. In summary, the added value in this area of this paper is the suggestion of a new MCDM method to solve the problems raised in the previous literature. To our knowledge, this is the first study trying to utilize integrated weighting approach and fuzzy VIKOR for evaluating mobile service. With the proposed method, service quality of mobile service can be assessed effectively, so it provides a new way for company to manage and evaluate mobile services’ demonstrated competence in the perspective of service management. In addition, a special advantage of VIKOR method is the practical application possibilities for unique and complex decision situations encountered when evaluating mobile services because with this approach we can compare evaluation results by adjusting influence levels of the value in VIKOR method.

The remainder of this paper is organized in the following order. In Section 2, we explain methodological background including the basic concepts of fuzzy sets, subjective and objective weights, and fuzzy VIKOR. Section 3 outlines the overall research framework and details the process steps. Section 4 provides an empirical case study to make it easier to understand the proposed approach. This section also conducts the sensitivity analysis of the proposed approach and validation analysis by comparing the results with other representative MCDM methods. Finally, in section 5, conclusions of the paper are provided and we anticipate future research.

## Methodological background

### Linguistic variables and fuzzy numbers

**Fig 1:**
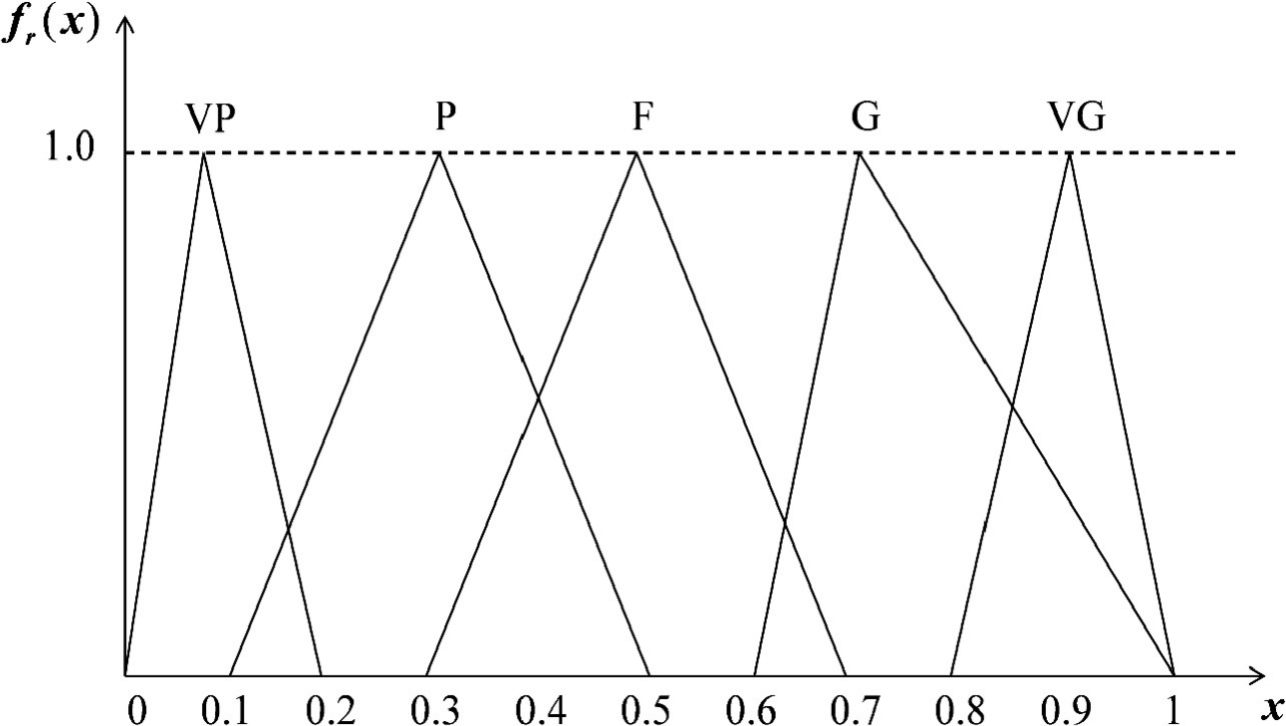
Membership functions of linguistic variables for measuring rating of alternatives.

According to Zadeh [39], traditional quantification methods are not suitable for expressing complex or difficult-to-define situations; This task claims language variables are needed [40–42]. In this study, five linguistic variables were utilized for calculating the ratings for alternative websites (see Fig 1). The fuzzy numbers suggested by Quang et al. [40], listed in Table 2, were incorporated into the computational technique.

**Table 2:** The TFNs of linguistic variables for rating of alternatives.

Linguistic variables	IVFNs
Very poor (VP)	(0.0, 0.1, 0.2)
Poor (P)	(0.1, 0.3, 0.5)
Fair (F)	(0.3, 0.5, 0.7)
Good (G)	(0.6, 0.7, 0.9)
Very good (VG)	(0.8, 0.9, 1.0)

**Fig 2:**
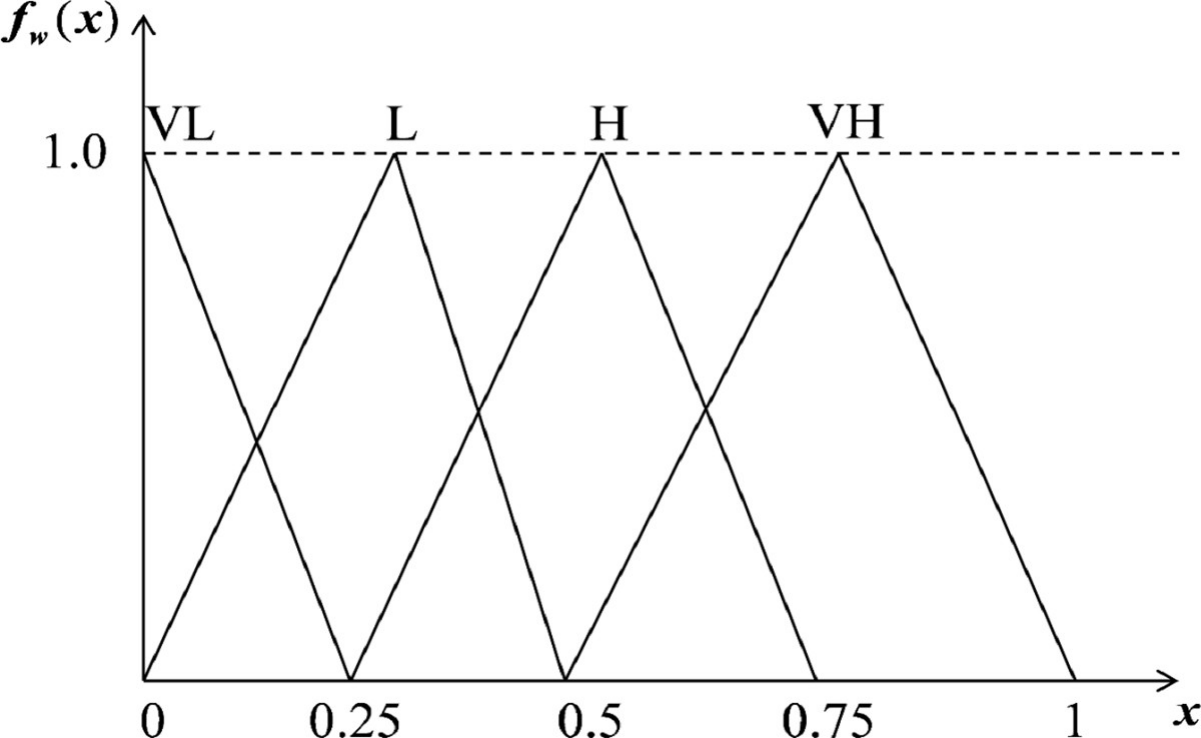
Membership functions of linguistic variables for the importance weight of criteria.

We also utilized linguistic variables for measuring the importance weights of each criterion (see Fig 2). The triangular fuzzy numbers (TFNs) suggested by Chou et al. [41] and Kang, Jang (43) are listed in Table 3.

**Table 3:** The TFNs of linguistic variables for the importance weight of criteria.

Linguistic variables	IVFNs
Very high influence (VH)	(0.5, 0.75, 1)
High influence (H)	(0.25, 0.5, 0.75)
Low influence (L)	(0, 0.25, 0.5)
Very low influence (VL)	(0, 0, 0.25)
No influence (N)	(0, 0, 0)

In order to use the language variable for evaluation, the defuzzification process must be performed to convert it into crisp value [43]. When a defuzzification process is conducted, the fuzzy number can be transformed as a specific crisp value called as a Best Non-fuzzy Performance (BNP) measure. The various defuzzification methods were suggested in previous research. This study utilized the graded mean integration representation method suggested by Chen and Wang [44] among various fuzzy methods to derive a single fuzzy number by applying the integral of graded mean h-level of fuzzy number because it has the simple calculation process and excludes analysts’ opinions. The ***BNP*** is calculated based on the following graded mean integration representation method:

$$\begin{array}{l} L_{A}(x)=(x-a)/(b-a),a\leq x\leq b,\;L_{A}^{-1}(h)=a+(b-a)h,0\leq h\leq 1\\ R_{A}(x)=(x-c)/(b-c),b\leq x\leq c,\;R_{A}^{-1}(h)=a+(b-c)h,0\leq h\leq 1 \end{array}$$ (1)

***L***_***A***_(***x***) and ***R***_***A***_(***x***) are the function ***L*** and the function ***R*** of the triangular fuzzy number ***A***, respectively. $L_{A}^{-1}(h)$ and $R_{A}^{-1}(h)$ are the inverse functions of the function ***L***_***A***_(***x***) and the function ***R***_***A***_(***x***) at ***h***-level, respectively. Let the graded mean ***h***-level value of fuzzy number ***A*** is $h(L_{A}^{-1}(h)+R_{A}^{-1}(h))/2$. Then, ***BNP*** calculated by the graded mean integration representation method of ***A*** is as follows:

$$\begin{array}{cc} \begin{array}{l} BNP=\int_{0}^{1}\frac{h(L_{A}^{-1}(h)+R_{A}^{-1}(h))}{2}dh/\int_{0}^{1}hdh\\ =\int_{0}^{1}\frac{h(a+(b-a)h+a+(b-c)h)}{2}dh/\int_{0}^{1}hdh\\ =\left\{\left(c-a\right)+\left(b-a\right)\right\}/3+a \end{array} & \;\mathrm{where}\;\tilde{A}=(a,b,c) \end{array}$$ (2)

### Subjective and objective weights

In MCDM problems, assessing the weights of criteria is an important issue. Weights of criteria should reflect the respective relative importance in the decision-making process [13]. It can not be assumed that each criterion has the same significance because the evaluation of weights of criteria involves a variety of opinions and meanings [45]. The existing approaches can be divided into two categories: the subjective approach and objective approach. As mentioned in introduction, subjective weighting approach is based on the decision maker (DM)s’ subjective preferences. In this approach, the weights of DMs and attributes are usually given in advance, or a special evaluation matrix is established to compare the differences of the DMs and attributes [46–48]. It can effectively reflect various DMs’ preferences. However, the subjective weighting method requires the experts to be very familiar with each other and the decision problem, but even so, the subjectivity and uncertainty is still strong [49]. Meanwhile, objective weighting approach is based on data which are given in the decision table of the attributes for each alternative [50, 51]. In this approach, the common element is that there is no need to have another evaluation matrix for the DMs and attributes, and the weights of the DMs and attributes are only computed by data which are given in the decision table for each alternative [52]. However, objective weighting method does not consider opinions of the experts for weighting criteria. Because these kinds of methods are more objective and accurate, it has resulted in wide research for several years [49]. To sum up, although both methods have their advantages and disadvantages, there is little research on the weighting of criteria by combining the subjective weighted approach and the objective weighted approach. In the actual decision situation, considering objective assessment information of decision opinions as well as the differences of the subjective preferences of DMs and their identity differences can make the decision result more accurate. Thus, this study utilizes integrated weighting approach based on both subjective and objective weights for weighting for criteria. For calculating subjective weights, DEMATEL is utlized in this study. DEMATEL can be used not only to transform the relationship between the cause and effect of the criterion into a visual structural model but also to process internal dependencies within a set of criteria and effectively calculate the weight of the criterion [53–56]. In terms of objective weights, Shannon’s entropy is utilized. This study adopted the concept of information entropy to identify the weight of evaluation attributes that can effectively balance the effects of subjective factors. The detailed calculation procedures for weighting criteria will be explained in Section 3.

#### Subjective weights: DEMATEL method

The DEMATEL method was originally presented by the Science and Human Affairs Program of the Battelle Memorial Institute of Geneva in 1972–1976, was used to solve complex and interrelated decision-making problems [57]. DEMATEL, as one of subjective weighting approaches, is the method that can be used to model causal dependencies among criteria. This method is able to visualize the complex cause and effect relationships in an understandable manner [58]. It is especially practical and useful for decision makers who want to analyze as well as graphically solve causal relationship problems based on matrices or digraphs in terms of three reasons [59]. First, digraphs of DEMATEL portray a contextual relation among the elements of the system by converting information about relationships among factors into visible structural model. Second, DEMATEL can clarify the strength of interactions between sub-systems. Lastly, based on information of “Prominence” and “Influence” derived from the strength of interactions between sub-systems, DEMATEL can provide the importance weight of criteria autonomously for evaluating system. In this study, a fuzzy DEMATEL method employing the fuzzy set theory is utilized as a subjective method.

#### Objective weights: Shannon entropy measure

The entropy concept was firstly proposed by Shannon et al. [60], which measures the uncertainty of information formulated in terms of probability theory. As is known, in the field of thermodynamics, we can measure the disorder in a system with entropy. Having been transferred from the field of thermodynamics to the information domain, Shannon entropy can be widely employed to evaluate the degree of disorder and the effectiveness of the information for a system [13].

Shannon proposed the *H* measure that satisfies all three of the following three properties for all *p*_*i*_ within the estimated joint probability distribution *P* [61]:

1. *H* is a continuous positive function;
2. If all *p*_*i*_ are equal, $p_{i}=\frac{1}{n}$, *H* should be a monotonically increasing function of *n*
3. For all *n*≥2, $H(p_{1},p_{2},\ldots ,p_{n})=H(p_{1}+p_{2},p_{3},\ldots ,p_{n})+(p_{1}+p_{2})H(\frac{p_{1}}{p_{1}+p_{2}},\frac{p_{2}}{p_{1}+p_{2}})$.

Shannon showed that the only function that satisfies these properties is

$$H(P)=-\sum_{i}p_{i}\log(p_{i})$$ (3)

This concept of Shannon’s has been well deployed as a weighting calculation method [13, 14, 62, 63]. In entropy measure, the smaller the entropy value, the smaller the degree of disorder in the system and the higher the weight [64]. In other words, the larger the value of the entropy, the smaller the entropy weight, and the smaller the different alternatives in this particular attribute, the less information the specific attribute provides, and the less important this attribute becomes in the decision-making process [13]. In this paper, we utilize the entropy measure as an objective weight.

### Fuzzy VIKOR method

The VIKOR method was developed for multicriteria optimization in the complex systems. VIKOR is a compromise ranking method for optimization of the multi-response process [65]. It introduces the multicriteria ranking indexes based on specific measures of “closeness” to the “ideal” solution [66]. The core of VIKOR lies in ranking and selection in the set of alternatives with conflicting criteria [67]. In VIKOR, the ranking index is derived by taking into account both the *maximum group utility* and the *minimum individual regret* of the opponent [6]. That is, assuming that each alternative can be evaluated by each criterion, the compromise ranking can be derived by comparing the measure of closeness to the ideal alternative.

VIKOR is a useful tool for multicriteria decision making in a situation where the decision makers are not able, or unaware of expressing their preferences at the beginning of system design [68]. The acquired compromise solution could be accepted because it provides a maximum “group utility” of the “majority”, and a minimum of the individual regret of the “opponent”. The compromise solutions can be the basis for negotiations and can include decision-maker's baseline weight preferences. Indeed, over the course of twenty years, much research has been conducted using the VIKOR method.

In recent years, a fuzzy VIKOR method has been proposed that uses fuzzy set theory for modeling the complex systems due to serious practical problems in applying VIKOR related to uncertainty of human cognition and ambiguous judgment of human perception [23, 69–72]. This is one of the key approaches used in this study.

## Proposed approach for evaluating mobile service

### Overall research framework

**Fig 3:**
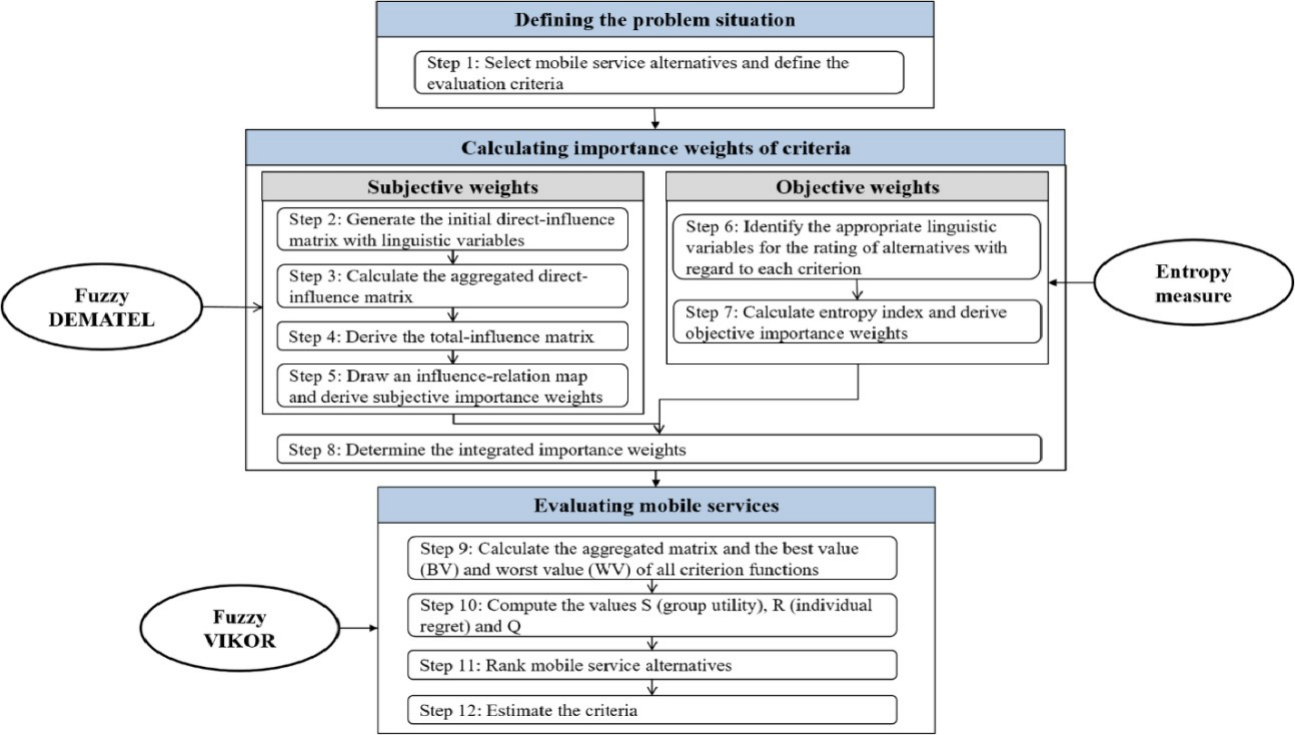
Overall research framework.

The outline of the proposed framework is shown in Fig 3. As shown, the whole process of study is made up of three main phases: *definition of the problem situation*, *calculation of importance weights*, and *evaluation of mobile services*; these three phases include sub-steps as a total of 11 detailed procedures.

At the phase of *defining the problem situation*, several mobile services that provide similar functionality are first selected as an alternative. Then, based on literature review and expert opinion, we define *service-based and product-based criteria* to reflect common characteristics of alternatives.

In the *calculating importance weights* phase, firstly, subjective weights for criteria are derived using fuzzy DEMATEL. To derive subjective weights for criteria, a total-influence matrix is configured to capture the impact level of each criterion by comparing it with the other criterion. Based on this, the data set is mapped to obtain the Influence Relationship Map (IRM) and the importance weight of the criterion is obtained. Secondly, objective weights for criteria are calculated using entropy measure. Here, entropy index is derived based on decision matrix, which consists of linguistic variables for the rating of alternative. Lastly, the integrated importance weights are determined based on the results of subjective and objective weights.

In the *evaluating mobile services* phase, the rating of the various service alternatives based on linguistic variables is calculated for each criterion and the fuzzy VIKOR method is used to calculate the S (group utility) and R (individual regret) values. Finally, consideration and adjustment of S and R values prioritize mobile service alternatives.

### Defining the problem situation

**Step 1**. **Select mobile service alternatives and define the evaluation criteria**

Mobile service alternatives are selected as the first step in evaluating mobile service. At this stage, it is usually important to choose mobile services that fall under the same category. This is because, in the same category of mobile services, the important characteristics that customers consider are not much different from other mobile services. Thus, from the same mobile service category, several mobile services can be selected as evaluation alternatives. The critical service-based and product-based evaluation criteria for selected mobile service alternatives can be derived based on the literature review and expert judgment.

### Calculating importance weights of criteria

**Step 2**: **Generate an initial direct-influence matrix using linguistic variables**

This study used a fuzzy DEMATEL method, as a subjective weighting approach, to portray the contextual relationships among the elements of the system, and finally, to obtain the criteria weights.

To measure causal relationships among criteria ***C*** = {***C***_***i***_|***i*** = 1,2,…,***n***}, a decision group of ***k*** experts is asked to demonstrate the direct influence, based on ***n*** x ***n*** matrix of pairwise comparison, as

$${\tilde{Z}}_{p}=\begin{array}{c} C_{1}\\ C_{2}\\ \ldots \\ C_{n} \end{array}\left[\begin{array}{cccc} 0 & {\tilde{z}}_{12p} & \ldots & {\tilde{z}}_{1np}\\ {\tilde{z}}_{21p} & 0 & \ldots & {\tilde{z}}_{2np}\\ \ldots & \ldots & \ldots & \ldots \\ {\tilde{z}}_{n1p} & {\tilde{z}}_{n2p} & \ldots & 0 \end{array}\right]$$ (4)

where ${\tilde{z}}_{ijp}=(a_{ijp},b_{ijp},c_{ijp})$ is the evaluation score of the ***p***th expert in the decision group consisting of ***k*** experts with triangular fuzzy numbers.

**Step 3**: **Calculate the aggregated direct-influence matrix**

In this step, the aggregated direct-influence matrix is constructed by averaging ***k*** defuzzified matrices that are transformed from ***k*** direct relation matrices containing fuzzy numbers. First, the defuzzified matrix based on the method of the BNP value is calculated using Eq (2) as

$$\begin{array}{cc} {\tilde{Y}}_{p}=\begin{array}{c} C_{1}\\ C_{2}\\ \ldots \\ C_{n} \end{array}\left[\begin{array}{cccc} {\tilde{y}}_{11p} & {\tilde{y}}_{12p} & \ldots & {\tilde{y}}_{1np}\\ {\tilde{y}}_{21p} & {\tilde{y}}_{22p} & \ldots & {\tilde{y}}_{2np}\\ \ldots & \ldots & \ldots & \ldots \\ {\tilde{y}}_{n1p} & {\tilde{y}}_{n2p} & \ldots & {\tilde{y}}_{nnp} \end{array}\right] & \mathrm{where}\;{\tilde{y}}_{ijp}=\left\{\left(c_{ijp}-a_{ijp})+(b_{ijp}-a_{ijp}\right)\right\}/3+a_{ijp} \end{array}$$ (5)

Then, the aggregated direct-influence matrix by averaging ***k*** defuzzified matrices is calculated as

$$\tilde{Y}=\frac{1}{k}\sum_{p=1}^{m}{\tilde{Y}}_{p}=\begin{array}{c} C_{1}\\ C_{2}\\ \ldots \\ C_{n} \end{array}\left[\begin{array}{cccc} {\tilde{y}}_{11} & {\tilde{y}}_{12} & \ldots & {\tilde{y}}_{1n}\\ {\tilde{y}}_{21} & {\tilde{y}}_{22} & \ldots & {\tilde{y}}_{2n}\\ \ldots & \ldots & \ldots & \ldots \\ {\tilde{y}}_{n1} & {\tilde{y}}_{n2} & \ldots & {\tilde{y}}_{nn} \end{array}\right]$$ (6)

Second, based on the aggregated direct-influence matrix, the normalized direct relation matrix X can be obtained by

$$\begin{array}{cc} \tilde{X}=\begin{array}{c} C_{1}\\ C_{2}\\ \ldots \\ C_{n} \end{array}\left[\begin{array}{cccc} {\tilde{x}}_{11} & {\tilde{x}}_{12} & \ldots & {\tilde{x}}_{1n}\\ {\tilde{x}}_{21} & {\tilde{x}}_{22} & \ldots & {\tilde{x}}_{2n}\\ \ldots & \ldots & \ldots & \ldots \\ {\tilde{x}}_{n1} & {\tilde{x}}_{n2} & \ldots & {\tilde{x}}_{nn} \end{array}\right] & \mathrm{where}\;{\tilde{x}}_{ij}=\frac{{\tilde{y}}_{ij}}{\sum_{j}\sum_{i}{\tilde{y}}_{ij}} \end{array}$$ (7)

**Step 4**: **Derive the total-influence matrix**

Once a normalized direct-influence matrix is obtained, the total-influence matrix can be calculated by the equations

$$\begin{array}{ll} \begin{array}{l} \tilde{T}=\tilde{X}+{\tilde{X}}^{2}+\ldots +{\tilde{X}}^{k}\\ =\tilde{X}\left(I+\tilde{X}+{\tilde{X}}^{2}+\ldots +{\tilde{X}}^{k-1}\right)\\ =\tilde{X}\left(I+\tilde{X}+{\tilde{X}}^{2}+\ldots +{\tilde{X}}^{k-1}\right)(I-\tilde{X}){\left(I-\tilde{X}\right)}^{-1}\\ =\tilde{X}{\left(I-\tilde{X}\right)}^{-1} \end{array} & \mathrm{when}\;\underset{k\to \infty }{\lim}{\tilde{X}}^{k}={\left[0\right]}_{n\times n} \end{array}$$ (8)

and

$$\tilde{T}=\begin{array}{c} C_{1}\\ C_{2}\\ \ldots \\ C_{n} \end{array}\left[\begin{array}{cccc} {\tilde{t}}_{11} & {\tilde{t}}_{12} & \ldots & {\tilde{t}}_{1n}\\ {\tilde{t}}_{21} & {\tilde{t}}_{22} & \ldots & {\tilde{t}}_{2n}\\ \ldots & \ldots & \ldots & \ldots \\ {\tilde{t}}_{n1} & {\tilde{t}}_{n2} & \ldots & {\tilde{t}}_{nn} \end{array}\right]$$ (9)

**Step 5**: **Draw influence relation map and derive importance weights**

The sums of the rows and columns of $\tilde{T}$ can be denoted by ***R*** and ***C***, respectively, as

$$R=[\sum_{j=1}^{n}t_{ij}{]}_{n\times 1}={\left[r_{i}\right]}_{n\times 1},\;C=[\sum_{i=1}^{n}t_{ij}{]}_{1\times n}^{'}={\left[c_{j}\right]}_{1\times n}^{'}={\left[c_{j}\right]}_{n\times 1}$$ (10)

Here, ***r***_***i***_ is the sum of *i*th row in matrix $\tilde{T}$. The value of ***r***_***i***_ indicates the total effect, both direct and indirect effects of criterion *i* on the other criteria, whereas ***c***_***j***_ is the sum of *j*th column in matrix $\tilde{T}$. The value of ***c***_***j***_ indicates total effects, both direct and indirect effects that criterion *j* has received from the others. Additionally, ***r***_***j***_+***c***_***j***_ is the degree of the central role that criterion *j* plays in the system. On the other hand, if ***r***_***j***_−***c***_***j***_ is positive, then criterion *j* influences other criteria and therefore belongs to the cause group; and if ***r***_***j***_−***c***_***j***_ is negative, it is influenced by others and, as such, it belongs to the effect group.

Based on the dataset (***r***_***j***_+***c***_***j***_, ***r***_***j***_−***c***_***j***_), an IRM can be drawn. The horizontal axis ***r***_***j***_+***c***_***j***_ is denoted the “Prominence,” and the vertical axis ***r***_***j***_−***c***_***j***_ the “Influence.” IRM allows decision makers to visualize complex causal relationships between criteria as structural models. In addition, the importance weight of the criterion can be obtained as follows.

$$w_{j}^{'}={\left[{\left(r_{j}+c_{j}\right)}^{2}+{\left(r_{j}-c_{j}\right)}^{2}\right]}^{1/2},\;W_{j}^{sub}=w_{j}^{'}/\sum_{j=1}^{n}w_{j}^{'}$$ (11)

**Step 6**: **Identify appropriate linguistic variables for the assessed alternatives in relation to each criterion**

In order to determine the objective weight by the entropy measure, the decision matrix should be derived as follows.

$$\begin{array}{l} \begin{array}{cccc} \;C_{1} & \;C_{2} & & \;C_{n} \end{array}\\ {\tilde{U}}_{p}=\begin{array}{c} A_{1}\\ A_{2}\\ \ldots \\ A_{m} \end{array}\left[\begin{array}{cccc} {\tilde{r}}_{11p} & {\tilde{r}}_{12p} & \ldots & {\tilde{r}}_{1np}\\ {\tilde{r}}_{21p} & {\tilde{r}}_{21p} & \ldots & {\tilde{r}}_{2np}\\ \ldots & \ldots & \ldots & \ldots \\ {\tilde{r}}_{m1p} & {\tilde{r}}_{m2p} & {\tilde{r}}_{m3p} & {\tilde{r}}_{mnp} \end{array}\right] \end{array}$$ (12)

where ${\tilde{r}}_{ijp}=(a_{ijp},b_{ijp},c_{ijp})$ is an evaluation rating of the ***p***th expert in a decision group consisting of ***k*** experts with triangular fuzzy numbers.

$$\begin{array}{l} \begin{array}{cccc} \;C_{1} & \;C_{2} & & \;C_{n} \end{array}\\ {\tilde{W}}_{p}=\begin{array}{c} A_{1}\\ A_{2}\\ \ldots \\ A_{m} \end{array}\left[\begin{array}{cccc} {\tilde{w}}_{11p} & {\tilde{w}}_{12p} & \ldots & {\tilde{w}}_{1np}\\ {\tilde{w}}_{21p} & {\tilde{w}}_{22p} & \ldots & {\tilde{w}}_{2np}\\ \ldots & \ldots & \ldots & \ldots \\ {\tilde{w}}_{m1p} & {\tilde{w}}_{m2p} & \ldots & {\tilde{w}}_{mnp} \end{array}\right]\;\mathrm{where}\;{\tilde{w}}_{ijp}=\{(c_{ijp}-a_{ijp})+(b_{ijp}-a_{ijp})\}/3+a_{ijp} \end{array}$$ (13)

Second, based on defuzzified matrices, the aggregated matrix can be calculated by averaging ***k*** matrices as

$$\begin{array}{l} \begin{array}{cccc} \;C_{1} & \;C_{2} & & \;C_{n} \end{array}\\ \tilde{F}=\frac{1}{k}\sum_{p=1}^{k}{\tilde{W}}_{p}=\begin{array}{c} A_{1}\\ A_{2}\\ \ldots \\ A_{m} \end{array}\left[\begin{array}{cccc} f_{11} & f_{12} & \ldots & f_{1n}\\ f_{21} & f_{22} & \ldots & f_{2n}\\ \ldots & \ldots & \ldots & \ldots \\ f_{m1} & f_{m2} & \ldots & f_{mn} \end{array}\right] \end{array}$$ (14)

**Step 7**: **Calculate entropy index and derive objective importance weights**

After deriving the aggregated decision matrix, the decision matrix needs to be normalized as

$${\left[p_{ij}\right]}_{m\times n}=[f_{ij}/\sum_{i=1}^{m}f_{ij}{]}_{m\times n}$$ (15)

We can calculate the entropy values *e*_*j*_ as

$$e_{j}=-k\sum_{i=1}^{m}p_{ij}\;\ln p_{ij}$$ (16)

where *k* is Boltzman’s constant, which equals *k* = (ln(*m*))^−1^.

The degree of diversification *div*_*i*_ of the intrinsic information of each criterion *C*_*j*_(*j* = 1,2,…,*n*) can be calculated as

$$div_{j}=1-e_{j}.$$ (17)

The value *div*_*j*_ represents the inherent contrast intensity of *C*_*j*_. Thus, the higher the *div*_*j*_ is, the more important the criterion *C*_*j*_ is to the problem. Finally, the objective weights for each criterion can be obtained as follows.

$$W_{j}^{Obj}=\frac{{div}_{j}}{\sum_{j}{div}_{j}}$$ (18)

**Step 8**: **Determine the integrated importance weights**

In consideration of both the objective and subjective weights, the integrated weights of the criteria are calculated as

$$W_{j}^{Integ}=\alpha W_{j}^{Sub}+(1-\alpha )W_{j}^{Obj}$$ (19)

where $W_{j}^{Integ}$ is the integrated weight of the jth criterion, and *α* and 1−*α* are coefficient values between 0 and 1 denoting the subjective and objective weights respectively.

### Evaluating mobiles services using fuzzy VIKOR

Fuzzy VIKOR denotes the various m alternatives as *A*_1_,*A*_2_,…,*A*_*m*_ as shown in Eq (12). For an alternative *A*_*i*_, the merit of the jth criterion is represented by *f*_*ij*_ and *f*_*ij*_ can be the value of the jth criterion function for the alternative *A*_*i*_, m being the number of criteria. Based on the derived decision matrix using Eqs (13) and (14), the fuzzy VIKOR procedure entails the following three steps:

**Step 9**: **Calculate the aggregated matrix and the best value (BV) and worst value (WV) of all criterion functions**

In this step, the best $f_{j}^{*}$ and worst $f_{j}^{-}$ values of all of the criterion functions are determined. If the jth criterion function represents a merit,

$$f_{j}^{*}=\max_{i}f_{ij}\;f_{j}^{-}=\min_{i}f_{ij}$$ (20)

**Step 10: Compute the values S**_**i**_ **(group utility), R**_**i**_ **(individual regret) and Q**_**i**_**, i = 1,2,3,…,m, by the relations**

$$S_{i}=\sum_{j=1}^{n}\frac{W_{j}^{Integ}(f_{j}^{*}-f_{ij})}{f_{j}^{*}-f_{j}^{-}}$$ (21)

and

$$R_{i}=\max_{j}[\frac{W_{j}^{Integ}(f_{j}^{*}-f_{ij})}{f_{j}^{*}-f_{j}^{-}}],$$ (22)

where *w*_*j*_ is the weight of the jth criterion that expresses the relative importances of the criteria, and

$$Q_{i}=v\left[\frac{S_{i}-S^{*}}{S^{-}-S^{*}}\right]+(1-v)\left[\frac{R_{i}-R^{*}}{R^{-}-R^{*}}\right]$$ (23)

where S* = min_*i*_*S*_*i*_, *S*^−^ = max_*i*_*S*_*i*_, R* = min_*i*_*R*_*i*_, *R*^−^ = max_*i*_*R*_*i*_, v is the weight of the *maximum group utility*, and (1−v) is the weight of the *individual regret*. Especially, if v is larger than 0.5, *Q*_*i*_ index follows the majority rule.

**Step 11**: **Rank mobile service alternatives**

As a final step of mobile service evaluation, the mobile service alternatives are ranked by sorting of the values S, R and Q in decreasing order. That is, the lower the Q value, the higher the preference.

**Step 12**: **Estimate the criteria**

The alternative *A*_1_ and *A*_2_ are, respectively, the alternative with first (minimum) and second positions in the ranking list by the measure *Q* (Minimum) if the following two conditions are satisfied [73–76].

- **C1**. Acceptable advantage:

$$Q(A_{2})-Q(A_{1})\geq DQ$$

where *Q*(*A*_2_) *and Q*(*A*_1_) are the first and second choice, respectively, and *m* is the number of alternatives

- **C2**. Acceptable stability in decision making

Especially, alternative *A*_1_ must also be the best ranked by *S* or/and *R*. In addition, if one of the conditions is not satisfied, then a set of compromise solutions is proposed, as follows.

- If only the condition C2 is not satisfied, alternatives *A*_1_ and *A*_2_ are compromise solutions.
- If condition C1 is not satisfied (*Q*(*A*_2_)−*Q*(*A*_1_)≥*DQ*), then alternatives *A*_1_,*A*_2_,…,*A*_*m*_ are considered as compromise solutions; *A*_*m*_ is determined by the relation *Q*(*A*_*m*_)−*Q*(*A*_1_)<*DQ* for maximum *M* (the positions of these alternatives are ‘‘in closeness”)

## Empirical case study on mobile service evaluation

### Empirical case study overview

To illustrate the utility of the proposed approach, empirical case study was conducted. In the mobile age, mobile services have proven to be a core service type, especially because customers use this service without the space and time constraints. The number of mobile services registered in the App Store, the mobile service market, is already over 500,000. There are also 20 mobile service categories, including books, business, finance, games, music, navigation, and social networking. This proliferation has made it difficult for customers to choose a specific mobile service from a range of services with similar capabilities in the same category. Therefore, current empirical case studies have focused on evaluating mobile services to help customers choose the best mobile services.

### Defining the problem situation

**Step 1**: **Select alternative mobile services and define the evaluation criteria**

Of the 20 mobile services categories in the App Store, the navigation category has been selected. Because there are many similar services in this category that have similar functionality to other service categories, so the navigation category was selected. In the navigation category, six mobile services widely used in Korea were selected as an alternative: *navigation service 1 (****A***_1_*)*, *navigation service 2 (****A***_2_*)*, *navigation service 3 (****A***_3_*)*, *navigation service 4 (****A***_*4*_*)*, *navigation service 5 (****A***_5_*)*, *and navigation service 6 (****A***_6_*)*.

Then, from the literature of mobile service and navigation service, candidate factors affecting the evaluation of mobile navigation service are figured out [34, 35, 37, 77–84]. We selected 4 experts regarding mobile service. 4 experts panel are comprised two mobile service developers and two heavy users of mobile navigation service. They specialize in service engineering in the field of technical management. In addition, these professionals are heavy users of mobile services, so they have a huge volume of information for evaluating mobile services. After discussion with 5 experts panel, as shown in Tables 4 and 8 factors are finalized as evaluation criteria for evaluating mobile navigation services with regard to both *service-based criteria* and *product-based criteria*.

**Table 4:** Criteria for mobile service evaluation within navigation category.

Type	Aspect and criterion	Description	References
*Service-based criteria*	Customized option (***C***_1_)	Degree of freedom for customizing option	[77]
	Update (***C***_2_)	Continuous updates for bug fixing, improvement of functions, and addition of new functions	[78]
	Search (***C***_3_)	Ease of searching of various information	[77, 79]
	Audio guidance (***C***_4_)	Ease of understanding of audio guidance	[80]
*Product-based criteria*	Speed (***C***_5_)	Responsiveness for assigned task	[34, 35]
	Display (***C***_6_)	Ease of understanding of visualized information	[36, 81]
	Connectivity (***C***_7_)	Ease of connection to network	[37, 82]
	Interface (***C***_8_)	Accessibility of functions through simple operations	[83, 84]

### Calculating importance weights of criteria

**Step 2**: **Generate the initial direct-influence matrix with variables**

A decision group composed of 4 experts was asked to indicate direct influence by measuring, based on an n x n pairwise comparison matrix, the casual relationships among 8 criteria. The levels of direct influence among the criteria were evaluated according to the following linguistic scale: no influence (N), very low influence (VL), low influence (NL), high influence (H), and very high influence (VH) (see Table 5).

**Table 5:** Initial direct-influence fuzzy matrix as assessed by decision makers.

Judges	Criteria	*C*_1_	*C*_2_	*C*_3_	*C*_4_	*C*_5_	*C*_6_	*C*_7_	*C*_8_
***D***_1_	***C***_1_	N	VL	VL	L	L	VH	H	L
	***C***_2_	VL	N	L	VL	L	L	H	L
	***C***_3_	VL	VH	N	VL	L	L	H	L
	***C***_4_	L	L	VL	N	H	H	H	VH
	***C***_5_	VL	L	L	L	N	L	H	H
	***C***_6_	H	H	L	L	L	N	H	VL
	***C***_7_	H	H	H	H	L	H	N	L
	***C***_8_	L	H	L	VH	H	L	L	N
***D***_2_	***C***_1_	N	L	VL	L	l	VH	H	N
	***C***_2_	L	N	L	L	H	L	VH	L
	***C***_3_	L	VH	N	N	VH	L	VL	VL
	***C***_4_	H	L	N	N	VH	VH	VH	H
	***C***_5_	N	N	N	L	N	L	H	VH
	***C***_6_	H	L	N	H	VH	N	H	N
	***C***_7_	L	L	N	VL	VH	L	N	L
	***C***_8_	N	N	N	H	H	L	VL	N
***D***_3_	***C***_1_	N	VL	VL	VL	L	VH	VH	L
	***C***_2_	VL	N	VL	VL	H	H	VH	L
	***C***_3_	VL	VH	N	VL	L	H	H	L
	***C***_4_	L	VL	VL	N	VH	VH	VH	VH
	***C***_5_	VL	H	VL	L	N	L	H	H
	***C***_6_	L	H	VL	L	VL	N	L	VL
	***C***_7_	L	H	VL	H	H	VH	N	VL
	***C***_8_	VL	H	VL	H	H	L	L	N
***D***_4_	***C***_1_	N	VL	VL	VL	H	VH	L	VL
	***C***_2_	VL	N	VL	VL	VL	VL	H	VL
	***C***_3_	VL	VH	N	VL	VL	VL	VH	VL
	***C***_4_	VL	L	VL	N	H	H	VH	VH
	***C***_5_	VL	VL	VL	VL	N	VL	H	H
	***C***_6_	VH	VL	VL	VL	H	N	H	L
	***C***_7_	L	VH	VL	VL	H	H	N	L
	***C***_8_	VL	VL	VL	VL	H	VL	VL	N

**Step 3**: **Calculate the aggregated direct-influence matrix**

The evaluation results of the four experts were aggregated by averaging the values of four defuzzified matrices based on Eqs (5) and (6). For example, ${\tilde{z}}_{211}$ (VL) is defuzzified as follows.

$$\begin{array}{l} {\tilde{z}}_{211}=\left(0,\;0,\;0.25\right)\\ {\tilde{y}}_{211}=\{(0.25-0)+(0-0)\}/3+0=\frac{1}{12} \end{array}$$

Averaging the values of 4 defuzzified matrices is calculated as follows.

$${\tilde{y}}_{21}=\frac{1}{4}*({\tilde{y}}_{211}+{\tilde{y}}_{212}+{\tilde{y}}_{213}+{\tilde{y}}_{214})=\frac{1}{4}*(\frac{1}{12}+\frac{1}{4}+\frac{1}{12}+\frac{1}{12})=0.125$$

Then, the normalized direct matrix was derived using Eq (7) (see Table 6). For instance, ${\tilde{x}}_{21}$ is calculated as follows.

$${\tilde{x}}_{21}=\frac{{\tilde{y}}_{21}}{\sum_{j}\sum_{i}{\tilde{y}}_{ij}}=\frac{0.125}{0.75}=0.167$$

**Table 6:** Aggregated initial direct-influence defuzzified matrix.

		Service-based criteria				Product-based criteria			
		*C*_1_	*C*_2_	*C*_3_	*C*_4_	*C*_5_	*C*_6_	*C*_7_	*C*_8_
Service-based criteria	***C***_1_	0.000	0.167	0.111	0.222	0.417	1.000	0.667	0.194
	***C***_2_	0.167	0.000	0.222	0.167	0.444	0.361	0.833	0.278
	***C***_3_	0.167	1.000	0.000	0.083	0.500	0.361	0.667	0.222
	***C***_4_	0.222	0.278	0.028	0.000	0.833	0.833	0.917	0.917
Product-based criteria	***C***_5_	0.083	0.278	0.139	0.278	0.000	0.278	0.667	0.750
	***C***_6_	0.667	0.444	0.139	0.361	0.528	0.000	0.583	0.139
	***C***_7_	0.417	0.667	0.222	0.389	0.667	0.667	0.000	0.278
	***C***_8_	0.139	0.361	0.139	0.611	0.667	0.278	0.222	0.000

**Step 4**: **Derive the total–influence matrix**

Based on the aggregated direct-influence matrix, the total-influence matrix was derived using Eqs (8) and (9) (see Table 6). In the matrix, a value of 0.5 or greater, shown in bold in Table 7, can be considered a high influence relationship.

**Table 7:** Total-influence matrix and subjective weights.

		Service-based criteria				Product-based criteria						
		*C*_1_	*C*_2_	*C*_3_	*C*_4_	*C*_5_	*C*_6_	*C*_7_	*C*_8_	*r*_*i*_	*r*_*j*_+*c*_*j*_	*r*_*j*_−*c*_*j*_
Service-based criteria	***C***_1_	0.451	0.304	**0.510**	0.383	0.280	**0.788**	0.432	0.187	3.335	6.977	-0.306
	***C***_2_	0.468	0.286	**0.637**	0.395	0.389	0.392	**0.573**	0.374	3.513	6.990	0.037
	***C***_3_	0.404	**0.941**	0.494	0.233	0.322	0.288	**0.528**	0.229	3.438	7.621	-0.745
	***C***_4_	0.341	0.166	0.379	0.250	0.433	0.379	0.263	**0.709**	2.920	6.339	-0.500
Product-based criteria	***C***_5_	0.332	0.430	**0.549**	**0.573**	0.161	0.284	0.415	**0.773**	3.518	6.367	0.669
	***C***_6_	**0.764**	0.422	**0.527**	0.433	0.330	0.205	0.415	0.226	3.321	6.412	0.230
	***C***_7_	**0.555**	**0.531**	**0.571**	0.427	0.363	0.468	0.000	0.305	3.220	6.100	0.341
	***C***_8_	0.328	0.397	**0.516**	**0.726**	**0.571**	0.287	0.252	0.408	3.485	6.696	0.274
	***c***_***j***_	3.642	3.477	4.183	3.419	2.849	3.091	2.879	3.211			
	$W_{j}^{Sub}$	***0*.*130***	***0*.*130***	***0*.*143***	***0*.*119***	***0*.*119***	***0*.*120***	***0*.*114***	***0*.*125***			

**Step 5**: **Draw influence relation map (IRM) and derive importance weights**

**Fig 4:**
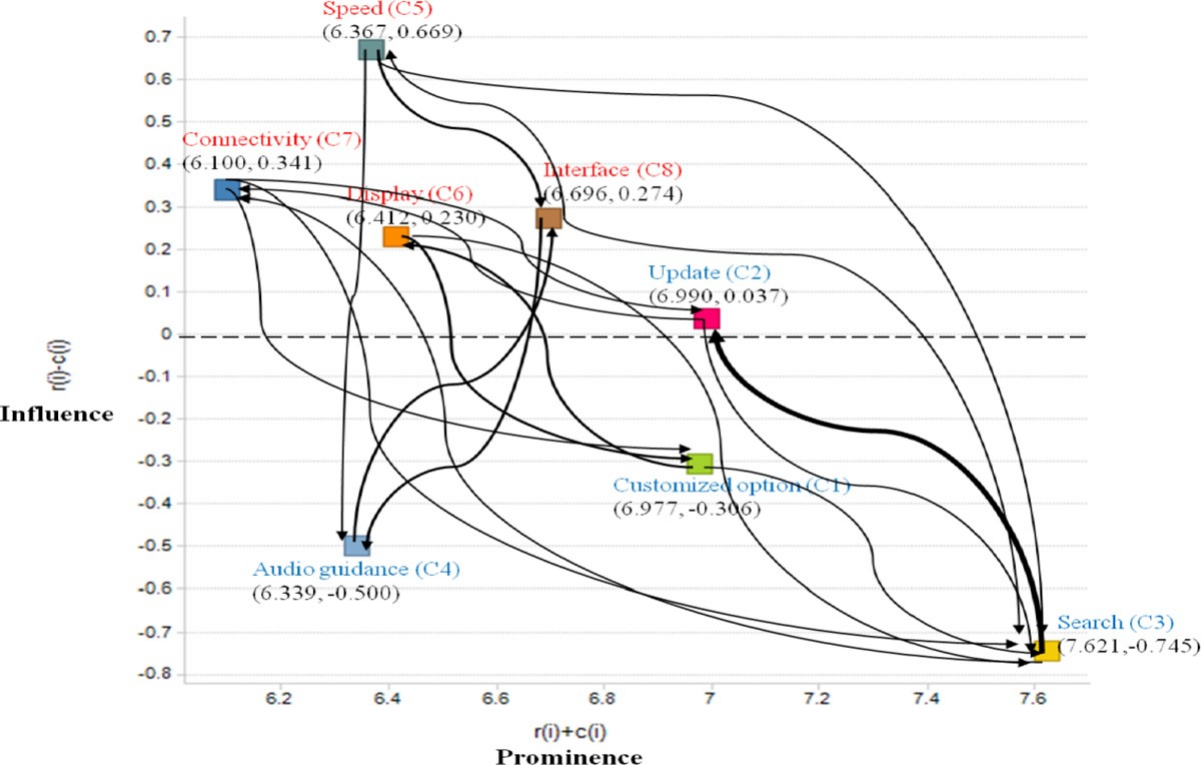
**Influence relation map (IRM)**.

At this step, the values of influence and relationship were calculated using Eq (10) (see Table 6). Based on the data set (***r***_***j***_+***c***_***j***_,***r***_***j***_−***c***_***j***_), the IRM is plotted as shown in Fig 4, where the influence relationship between the criteria is indicated by an arrow with a value of 0.5 or higher in Table 6. Higher values are indicated by thick arrows. According to the horizontal axis ***r***_***j***_+***c***_***j***_ (“Prominence”) and the vertical axis ***r***_***j***_−***c***_***j***_ (“Influence”), the criteria having positive ***r***_***j***_−***c***_***j***_ values belong to the cause group, whereas criteria having negative ***r***_***j***_−***c***_***j***_ values belong to the effect group. Finally, the importance weights of the criteria can be derived using Eq (11) (see Table 7).

As shown in the IRM (Fig 4), the evaluation criteria were visually divided into the cause group, which included update (***C***_2_), speed (***C***_5_), display (***C***_6_), connectivity (***C***_7_), and interface (***C***_8_), and the effect group, which included customized option (***C***_1_), search (***C***_3_), and audio guidance (***C***_4_). Speed (***C***_5_), with the highest value of ***r***_***j***_−***c***_***j***_, affected most of other criteria, as speed is a key attribute essential to the overall navigation performance of mobile services. Meanwhile, search (***C***_3_), with the highest value of ***r***_***j***_+***c***_***j***_, was most related to the other criteria and was the most influenced by all other criteria; furthermore, it was the most important one in the set of criteria with the highest weighting 0.143, as shown in Table 6.

In relation to the components of the causal group, the cause group is mainly composed of *product-based criteria*, excluding update (***C***_2_), which is a *service-based criteria*, whereas most of *service-based criteria*, such as option (***C***_1_), search (***C***_3_), and audio guidance (***C***_4_) belong to the effect group. From this result, in this particular navigation case, we inferred that the *product-based criteria* generally tended to affect the *service-based criteria*.

**Step 6**: **Identify the appropriate linguistic variables for the rated alternatives with regard to each criterion**

The decision matrix is constructed, then the defuzzified matrix is calculated using Eqs (12) and (13) as shown in Table 8. Based on the defuzzified matrices, the aggregated matrix is computed by averaging ***k*** matrices.

**Table 8:** Linguistic variables for rating of alternatives assessed by decision makers.

Judges	Candidates	Criteria							
		*C*_1_	*C*_2_	*C*_3_	*C*_4_	*C*_5_	*C*_6_	*C*_7_	*C*_8_
***D***_1_	*A*_1_	F	G	P	G	G	F	VG	F
	*A*_2_	G	P	G	F	VG	P	F	G
	*A*_3_	VG	G	G	P	P	VG	F	F
	*A*_4_	G	G	F	F	VP	F	P	G
	*A*_5_	G	VG	P	P	F	G	VG	G
	*A*_6_	P	P	G	F	F	VG	G	P
***D***_2_	*A*_1_	G	VG	P	F	G	P	G	F
	*A*_2_	G	F	VG	P	G	P	G	F
	*A*_3_	G	F	F	VP	F	G	G	G
	*A*_4_	VG	P	F	G	P	VP	F	VG
	*A*_5_	F	G	F	F	G	F	G	G
	*A*_6_	VP	VP	G	F	G	G	F	F
***D***_3_	*A*_1_	F	G	F	P	F	G	VG	P
	*A*_2_	F	P	G	G	VG	F	G	G
	*A*_3_	VG	G	P	F	G	VG	P	F
	*A*_4_	G	VG	F	F	G	VG	P	G
	*A*_5_	F	G	G	F	VG	P	P	VG
	*A*_6_	P	F	G	G	G	F	P	G
***D***_4_	*A*_1_	G	G	VG	F	P	F	G	G
	*A*_2_	VG	G	G	F	G	VG	F	G
	*A*_3_	G	F	G	P	F	F	VG	F
	*A*_4_	F	VP	G	F	G	G	VP	G
	*A*_5_	G	G	F	G	VG	G	F	G
	*A*_6_	F	P	G	G	VG	F	G	F

**Step 7**: **Calculate entropy index and derive objective importance weights**

Based on the aggregated decision matrix, the decision matrix needs to be normalized for calculating entropy index using Eq (15). Then, entropy index can be calculated using Eqs (16) and (17). Lastly, the objective weight for each criterion can be obtained using Eq (18) as shown in Table 9.

**Table 9:** Objective weights based on entropy measure.

	Entropy value (*e*_*j*_)	Degree of diversification (*div*_*i*_)	Objective weights ($W_{j}^{Obj}$)
***C***_**1**_	0.979	0.021	0.185
***C***_**2**_	0.974	0.026	0.227
***C***_**3**_	0.991	0.009	0.074
***C***_**4**_	0.988	0.012	0.102
***C***_**5**_	0.988	0.012	0.104
***C***_**6**_	0.994	0.006	0.055
***C***_**7**_	0.980	0.020	0.176
***C***_**8**_	0.991	0.009	0.079

**Step 8**: **Determine the integrated importance weights**

Based on both the objective and subjective weights, the integrated weights of the criteria are calculated using Eq (19) as shown in Table 10. In this case, we set ***α*** = 0.5 to reflect the objective and subjective weights equally.

**Table 10:** Integrated weights of criteria.

	Subjective weights ($W_{j}^{Sub}$)	Objective weights ($W_{j}^{Obj}$)	Integrated weights ($W_{j}^{Integ}$)
***C***_**1**_	0.130	0.185	0.157
***C***_**2**_	0.130	0.227	0.178
***C***_**3**_	0.143	0.074	0.108
***C***_**4**_	0.119	0.102	0.110
***C***_**5**_	0.119	0.104	0.111
***C***_**6**_	0.120	0.055	0.087
***C***_**7**_	0.114	0.176	0.145
***C***_**8**_	0.125	0.079	0.102

### Evaluation of mobile service

**Step 9**: **Calculate the aggregated matrix and the best value (BV) and worst value (WV) of all criterion functions**

First, the evaluation results of the four experts are averaged by the values of the defuzzified matrices based on Eqs (13) and (14), as shown in Table 11. Then, the BV and WV were calculated using Eq (20) as shown in Table 12.

**Table 11:** Aggregated defuzzified ratings of alternatives.

	*C*_1_	*C*_2_	*C*_3_	*C*_4_	*C*_5_	*C*_6_	*C*_7_	*C*_8_
*A*_1_	0.617	0.775	0.500	0.508	0.567	0.508	0.817	0.508
*A*_2_	0.717	0.458	0.775	0.508	0.817	0.500	0.617	0.675
*A*_3_	0.817	0.617	0.567	0.300	0.508	0.758	0.608	0.558
*A*_4_	0.717	0.508	0.558	0.558	0.467	0.558	0.300	0.775
*A*_5_	0.617	0.775	0.508	0.508	0.758	0.567	0.608	0.775
*A*_6_	0.300	0.300	0.733	0.617	0.717	0.658	0.567	0.508
*Weight* ($W_{j}^{Integ}$)	*0*.*157*	*0*.*178*	*0*.*108*	*0*.*110*	*0*.*111*	*0*.*087*	*0*.*145*	*0*.*102*

**Table 12:** Positive ideal solutions $f_{j}^{*}$ and negative ideal solutions $f_{j}^{-}$.

	*C*_1_	*C*_2_	*C*_3_	*C*_4_	*C*_5_	*C*_6_	*C*_7_	*C*_8_
$f_{j}^{*}$	0.817	0.775	0.775	0.617	0.817	0.758	0.817	0.775
$f_{j}^{-}$	0.300	0.300	0.500	0.300	0.467	0.500	0.300	0.508

**Step 10**: **Compute the values *S*_*i*_ (group utility), *R*_*i*_ (individual regret) and *Q*_*i*_**

First, based on results of Tables 11 and 12, the $W_{j}^{Integ}(f_{j}^{*}-f_{ij})/(f_{j}^{*}-f_{j}^{-})$ scores were calculated by applying weight ($W_{j}^{Integ}$) as shown in Table 13.

**Table 13:** Scores of $w_{j}\left(f_{j}^{*}-f_{ij}\right)/\left(f_{j}^{*}-f_{j}^{-}\right)$.

	*C*_1_	*C*_2_	*C*_3_	*C*_4_	*C*_5_	*C*_6_	*C*_7_	*C*_8_
*A*_1_	0.061	0.000	0.108	0.038	0.080	0.085	0.000	0.102
*A*_2_	0.030	0.119	0.000	0.038	0.000	0.087	0.056	0.038
*A*_3_	0.000	0.059	0.082	0.110	0.098	0.000	0.058	0.083
*A*_4_	0.030	0.100	0.085	0.020	0.111	0.068	0.145	0.000
*A*_5_	0.061	0.000	0.105	0.038	0.019	0.065	0.058	0.000
*A*_6_	0.157	0.178	0.016	0.000	0.032	0.034	0.070	0.102

Second, the values ***S***_***i***_ (group utility) and ***R***_***i***_ (individual regret), which can be between 0 and 1, were computed using Eqs (16) and (17) (see Table 14). Finally, based on these ***S***_***i***_ and ***R***_***i***_ values, ***Q***_***i***_ values were calculated using Eq (18). Here, the ***Q***_***i***_ values of each service were calculated using each ***v*** value as ***v*** = 0.5.

**Table 14:** *Q* values and ranking.

	*S*_*i*_	*R*_*i*_	*Q*_*i*_ (*v* = 0.5)	Rank
*A*_1_	0.473	0.108	0.284	3
*A*_2_	0.369	0.119	0.141	2
*A*_3_	0.491	0.110	0.333	4
*A*_4_	0.560	0.145	0.710	5
*A*_5_	0.346	0.105	0.000	1
*A*_6_	0.590	0.178	1.000	6

**Step 11**: **Rank alternatives of mobile services**

At this step, the priorities for the mobile service alternatives were determined as shown in Table 14. The alternatives were ranked by sorting the values ***S***, ***R*** and ***Q*** in decreasing order.

**Step 12**: **Estimate the criteria**

According to Acceptable stability in decision making (C2), alternative A_5_ must be the best ranked by S or/and R. This condition C2 is satisfied.

However, C1 is not satisfied because

$$Q(A_{1})=Q_{A5}=0\;Q(A_{2})=Q_{A2}=0.141$$

where v = 0.5

$$\begin{array}{l} Q(A_{2})-Q(A_{1})\geq \mathrm{DQ}=1/(m‐1)\\ \;0.141-0\geq \left(\frac{1}{6-1}\right)\gg \;0.141<0.20 \end{array}$$

The condition C1 is not satisfied (*Q*(*A*_2_)−*Q*(*A*_1_)≥*DQ*); then alternatives *A*_1_,*A*_2_,…,*A*_*m*_ are considered as compromise solutions; *A*_*m*_ is determined by the relation *Q*(*A*_*m*_)−*Q*(*A*_1_)<(*DQ* = 0.20) for maximum *M* (the positions of these alternatives are ‘‘in closeness”).

$$Q(A_{m})-(Q_{A5}=0)<(DQ=0.20)$$

The ranking based on *Q* is

$$A_{5}\approx A_{2}>A_{1}>A_{3}>A_{4}>A_{6}$$

The proposed model can provide additional information. If the ***v*** value is 0, the $v[\frac{S_{i}-S^{*}}{S^{-}-S^{*}}]$ term in Eq (23) becomes 0. This means that the ***Q***_***i***_ value is only affected by the ***R***_***i***_ value. This fact indicates which criterion should be considered the most essential to mobile service quality improvement. This provides guidance for identifying areas for improvement in certain aspects of service operations. For instance, with reference to Table 12 and the ***R***_***i***_ values in Table 13, most mobile services should focus on improvement of Update (***C***_2_), search (***C***_3_), Audio guidance (***C***_4_), and Connectivity (***C***_7_) service performance. On the other hand, Display (***C***_6_) and interface (***C***_8_) do not require much improvement in most services. Also, although *Navigation service 2 (****A***_5_*)* showed high service quality for all of the criteria, search (***C***_3_) could still be improved.

### Comprehensive discussion on the results

Six alternatives of mobile navigation service were evaluated using the proposed approach. To maximize the potential of this study, we need to consider two more issues related to the proposed approach. First, in the evaluation of mobile service, it is important to consider influence levels of coefficient (*α*) of integrated weight in Eq (19) and influence levels of the *v* value in VIKOR method, two types of sensitivity analysis should be conducted. Sensitivity analysis is conducted to investigate the influence levels of coefficient (*α*) of integrated weight. Then, sensitivity analysis for influence levels of the *v* value in VIKOR method can be progressed to know which alternatives are affected significantly by *maximum group utility* and *minimum individual regret*. Second, to verify the robustness of the proposed approach in this study, it is necessary to compare the results of the analysis with those of other methods. Validation by comparison with other major MCDM methods can demonstrate the advantages of the proposed approach. Sensitivity analysis and validation of results are therefore performed.

#### Sensitivity analysis

**Fig 5:**
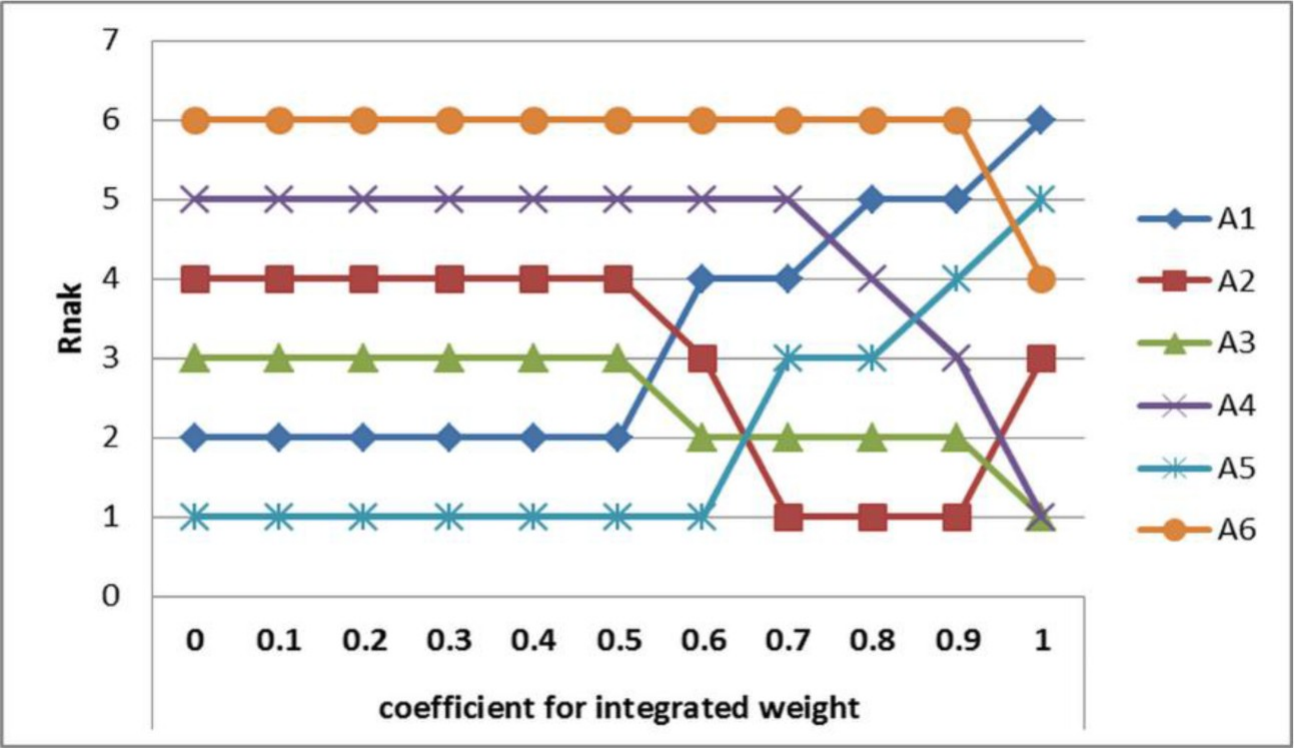
Sensitivity analysis of coefficient for integrated weight.

First, a sensitivity analysis of coefficient for integrated weight is conducted to investigate the influence levels of subjective and objective weights. The aim of sensitivity analysis is to observe the ranking order when the coefficient of subjective weights changes. The results of the sensitivity analysis are shown in Fig 5. When the coefficient value (*α*) was 0.5 or less, the rankings of alternatives were not at all affected. However, when the coefficient value (*α*) was 0.6 or more, the rankings of alternatives were changed. The rankings of ***A***_1_ and ***A***_5_ are decreased, whereas the rankings of ***A***_3_, ***A***_4_ and ***A***_6_ are increased. This fact reveals that the rankings of ***A***_1_ and ***A***_5_ is high when one focuses on objective weights. The rankings of ***A***_3_, ***A***_4_ and ***A***_6_ were high when coefficient value (*α*) was large and rises when the importance of the subjective weight increased. In other words, they scored higher service quality levels when subjective weights assessed by experts were considered to be important.

**Fig 6:**
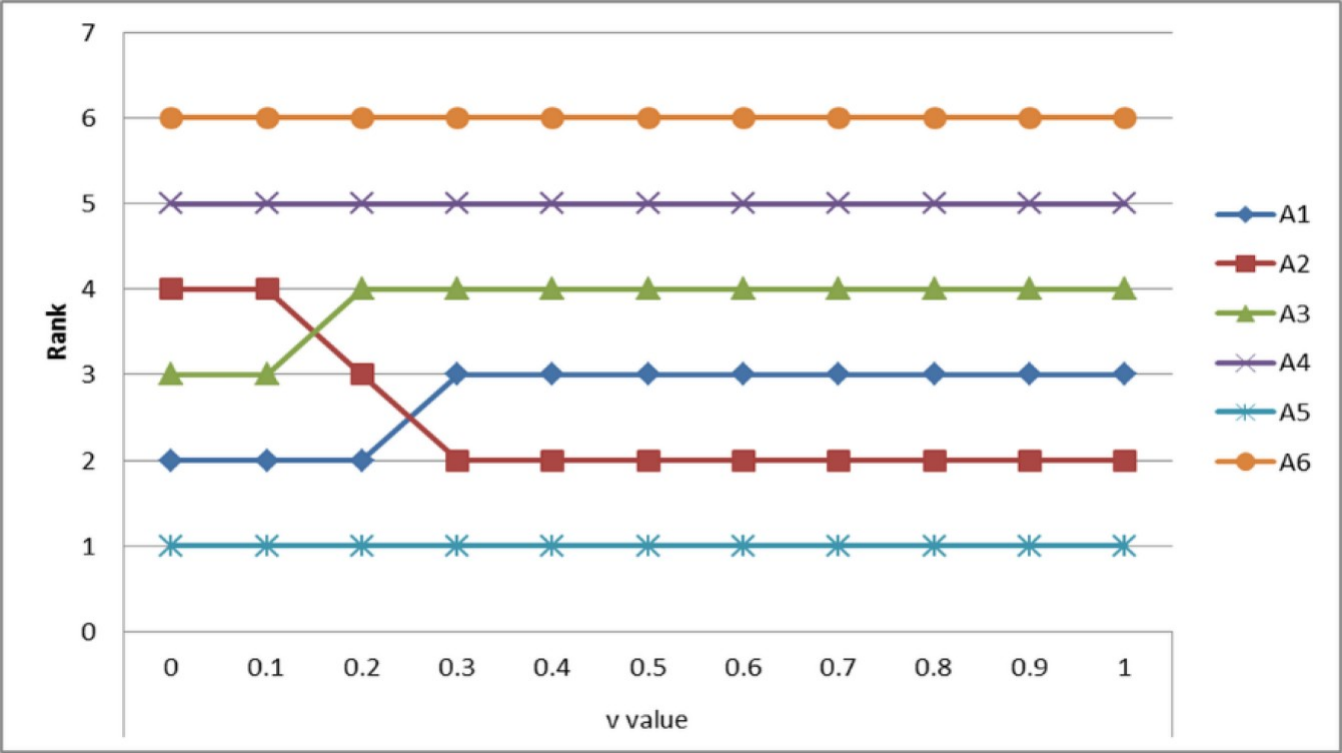
Sensitivity analysis for influence levels of the v value in VIKOR method.

Next, another sensitivity analysis is performed to determine the influence level of the v value with regard to the ranking order of the service alternatives (see Fig 6). ***A***_5_ was not affected by the v value. This means that ***A***_5_ has higher quality of service than other mobile services given the *maximum group utility* and *minimum individual regret*. Similarly, ***A***_4_ and ***A***_6_ was not affected by the v value. Indeed, these mobile services provide low service quality from the perspective of *maximum group utility* and *minimum individual regret*. On the other hand, the ranking of ***A***_2_ was improved according to increase of the v value. This reflects the fact that ***A***_2_ has been improved indicates that service quality has improved when focusing on *minimum individual regret*. In addition, the rankings of ***A***_1_ and ***A***_3_ were improved according to decreases of the v value. This indicates that ***A***_1_ and ***A***_3_ has a higher service quality when focusing on *maximum group utility*. That is, when *maximum group utility* was considered important, they recorded a high level of service quality.

#### Validation of results

We compared the results with the proposed approach by applying other representative MCDM methods to the same data for the validation test. For the validation, importance weights of criteria derived from integrated weight approach is applied to other MCDM methods. Here, we set *α* = 0.5 to reflect the objective and subjective weights equally. Table 15 shows that the results derived by applying the proposed approach are somewhat similar to those obtained using the other methods. The results of upper ranking group (***A***_1_, ***A***_2_, ***A***_5_) and lower ranking group (***A***_3_, ***A***_4_, ***A***_6_) were the same in all MCDM methods. However, the detailed rankings of alternatives were different for each MCDM method. The reason for this result is that other MCDM methods such as TOPSIS and GRA did not reflect both *maximum group utility* and *minimum individual regret* in the VIKOR method. However, the proposed approach effectively considered *maximum group utility* and the minimum *individual regret* of the opponent in the mobile service evaluation. Therefore, more accurate results can be obtained by using the proposed approach compared with other MCDM methods.

**Table 15:** Comparison with other methods.

MCDM	Ordering
Proposed method (In case of *v* = 0.5)	A5 ≈ A2 > A1 > A3 > A4 > A6
Grey relational analysis	A5 > A2 > A1 > A3 > A4 > A6
TOPSIS	A1 > A5 > A2 > A3 > A4 > A6

## Conclusions

This study presents a new MCDM approach by developing fuzzy VIKOR based on both subjective and objective weights for evaluation of mobile service quality. We combined the techniques of integrated weighting approach and fuzzy VIKOR, which together entail three main, consecutive phases. In the *defining the problem situation* phase, the problem situation is defined. In detail, mobile services are selected as evaluation alternatives, and the evaluation criteria are identified. In the *calculating importance weights* phase, importance weights of criteria are calculated using subjective and objective method. In particular, we use DEMATEL as a subjective weighting method to capture the influence level of each criterion on the other criteria, then an influence relation map (IRM) is derived by mapping the dataset. Lastly, the importance weights of the criteria are obtained. As an objective weight method, Shannon entropy, which is well known as an objective approach for identifying the weights of evaluation criteria, is utilized. Based on these two weight methods, integrated weights of criteria are derived in this stage. In the *evaluating mobile services* stage, the ratings of several mobile service alternatives are calculated for each criterion using the fuzzy VIKOR equations. Finally, sensitivity analysis and validation test with other MCDM methods were performed to verify the robustness of the proposed approach and the reliability of the results.

In order to utilize proposed framework, it is needed to gather two types of information: the causal relationships among criteria and rating of alternatives for each criterion. Information of the causal relationships among criteria is utilized to derive the subjective weights of criteria through DEMATEL method. Meanwhile, information of rating of alternatives for each criterion is utilized to calculate the objective weights of criteria using entropy measure. This information also is utilized for evaluating mobile services using VIKOR. In addition, in VIKOR method, weights of *maximum group utility* and the minimum *individual regret* of the opponent should be considered importantly. Thus, analysts need to perform a sensitivity analysis to evaluate the preference order according to the weight changes of *maximum group utility* and the minimum *individual regret*.

There are three contributions and potential utility of this approach. First, in order to overcome the limitations of the previous research focusing on mobile service evaluation with MCDM methods, we proposed a fuzzy VIKOR based on integrated weighting approach. One of the most important strengths of this new method is that it can adjust the rate of reflection for two types of weighting approaches based on decision makers’ own responsibilities taking into account the various characteristics of evaluation environment. Second, it uses fuzzy logics to effectively cover the uncertainty and the vagueness in mobile service evaluations. This can be the basis for further research dealing with the evaluation of mobile services. Third, fuzzy VIKOR can evaluate mobile service alternatives and analyze gaps in the desired level of performance for each mobile service systematically under ambigous and uncertain evaluation environment.

As a result of the empirical research, we have found that the proposed approach is a practical and efficient tool for evaluating mobile services in terms of their overall performance with regard to multiple criteria. The results of the comparison with other methods show that the proposed approach is consistent with the other methods. Based on the results of the verification of the empirical case and the comparison with other methods, the proposed approach was found to be effective and practical for solving the mobile service evaluation problem in which various evaluation criteria have complex causal relationships and influence each other. In addition, the important criteria for evaluating navigation mobile service could acquire a lot of attention in practical application. Moreover, the proposed approach was more reasonable than the other methods. Thus, this method can be applied to performance evaluation in other service sectors which have similar characteristics compared with those of mobile service industry. The proposed approach also provides guidance for identifying areas for improvement in certain aspects of service operations.

Despite all the strengths and applicability of the proposed method, there are some limitations to suggesting a path for future research. First, this study only considered 8 criteria for evaluating mobile navigation service in empirical case study. Future studies could apply the proposed approach based on considering much more criteria including economic and social criteria. Second, future study will integrate other significant impact criteria considering the hierarchical structure of criteria by using hierarchical MCDM approach. Third, by integrating the proposed approach into a computer-aided decision support system, an automated evaluation system can be constructed. Fourth, the kind of case study conducted in the current study should be performed for additional mobile services as well. Lastly, other advanced fuzzy logics such as Pythagorean fuzzy sets can be utilized to handle more uncertainty and various fuzzy logics can be compared to analyze the effectiveness.

## References

[1] Kim C-S, Oh E-H, Yang KH, Kim JK. The appealing characteristics of download type mobile games. Service Business. 2009;4(3):253–69. 10.1007/s11628-009-0088-0

[2] Kim J, Park Y, Kim C, Lee H. Mobile application service networks: Apple’s App Store. Service Business. 2014;8(1):1–27. 10.1007/s11628-013-0184-z

[3] Suh Y, Lee H, Park Y. Analysis and visualisation of structure of smartphone application services using text mining and the set covering algorithm: a case of App Store. International Journal of Mobile Communications. 2011;10(1):1–20.

[4] Azad N, Hashemi S. A study on important factors influencing customer relationship management: A case study of Mobile service provider. Management Science Letters. 2013;3(4):1161–6.

[5] Qi J-Y, Zhou Y-P, Chen W-J, Qu Q-X. Are customer satisfaction and customer loyalty drivers of customer lifetime value in mobile data services: a comparative cross-country study. Information Technology and Management. 2012;13(4):281–96. 10.1007/s10799-012-0132-y

[6] Kang D, Park Y. Review-based measurement of customer satisfaction in mobile service: Sentiment analysis and VIKOR approach. Expert Systems with Applications. 2014;41(4):1041–50.

[7] Büyüközkan G. Determining the mobile commerce user requirements using an analytic approach. Computer Standards & Interfaces. 2009;31(1):144–52. 10.1016/j.csi.2007.11.006

[8] Nikou S, Mezei J. Evaluation of mobile services and substantial adoption factors with Analytic Hierarchy Process (AHP). Telecommunications Pol. 2013;37(10):915–29. 10.1016/j.telpol.2012.09.007

[9] Shieh L-F, Chang T-H, Fu H-P, Lin S-W, Chen Y-Y. Analyzing the factors that affect the adoption of mobile services in Taiwan. Technol Forecast Soc Chang. 2014;87:80–8. 10.1016/j.techfore.2013.11.004

[10] Chen P-T, Cheng JZ. Unlocking the promise of mobile value-added services by applying new collaborative business models. Technol Forecast Soc Chang. 2010;77(4):678–93. 10.1016/j.techfore.2009.11.007

[11] Jyh-Fu Jeng D, Bailey T. Assessing customer retention strategies in mobile telecommunications: Hybrid MCDM approach. Management Decision. 2012;50(9):1570–95.

[12] Lu M-T, Tzeng G-H, Cheng H, Hsu C-C. Exploring mobile banking services for user behavior in intention adoption: using new hybrid MADM model. Service business. 2015;9(3):541–65.

[13] Wang T-C, Lee H-D. Developing a fuzzy TOPSIS approach based on subjective weights and objective weights. Expert Systems with Applications. 2009;36(5):8980–5. 10.1016/j.eswa.2008.11.035

[14] Shemshadi A, Shirazi H, Toreihi M, Tarokh MJ. A fuzzy VIKOR method for supplier selection based on entropy measure for objective weighting. Expert Systems with Applications. 2011;38(10):12160–7.

[15] Li Y, Zhu L. Optimization of user experience in mobile application design by using a fuzzy analytic-network-process-based Taguchi method. Applied Soft Computing. 2019;79:268–82. 10.1016/j.asoc.2019.03.048

[16] Ribeiro Soriano D, Jyh-Fu Jeng D, Bailey T. Assessing customer retention strategies in mobile telecommunications: Hybrid MCDM approach. Management Decision. 2012;50(9):1570–95.

[17] Lu M-T, Tzeng G-H, Cheng H, Hsu C-C. Exploring mobile banking services for user behavior in intention adoption: using new hybrid MADM model. Service Business. 2014;9(3):541–65. 10.1007/s11628-014-0239-9

[18] Gou X, Xu Z, Liao H. Hesitant fuzzy linguistic entropy and cross-entropy measures and alternative queuing method for multiple criteria decision making. Information Sciences. 2017;388–389:225–46. 10.1016/j.ins.2017.01.033

[19] Mohsen O, Fereshteh N. An extended VIKOR method based on entropy measure for the failure modes risk assessment–A case study of the geothermal power plant (GPP). Saf Sci. 2017;92:160–72.

[20] Chou S-Y, Chang Y-H, Shen C-Y. A fuzzy simple additive weighting system under group decision-making for facility location selection with objective/subjective attributes. European Journal of Operational Research. 2008;189(1):132–45.

[21] Lee H-C, Chang C-T. Comparative analysis of MCDM methods for ranking renewable energy sources in Taiwan. Renewable and Sustainable Energy Reviews. 2018;92:883–96. 10.1016/j.rser.2018.05.007

[22] Wang H, Jiang Z, Zhang H, Wang Y, Yang Y, Li Y. An integrated MCDM approach considering demands-matching for reverse logistics. Journal of Cleaner Production. 2019;208:199–210. 10.1016/j.jclepro.2018.10.131

[23] Narayanamoorthy S, Geetha S, Rakkiyappan R, Joo YH. Interval-valued intuitionistic hesitant fuzzy entropy based VIKOR method for industrial robots selection. Expert Systems with Applications. 2019;121:28–37. 10.1016/j.eswa.2018.12.015

[24] Wang Q, Wu C, Sun Y. Evaluating corporate social responsibility of airlines using entropy weight and grey relation analysis. Journal of Air Transport Management. 2015;42:55–62. 10.1016/j.jairtraman.2014.08.003

[25] Khademi-Zare H, Zarei M, Sadeghieh A, Saleh Owlia M. Ranking the strategic actions of Iran mobile cellular telecommunication using two models of fuzzy QFD. Telecommunications Pol. 2010;34(11):747–59. 10.1016/j.telpol.2010.10.001

[26] Deng H, Yeh C-H, Willis RJ. Inter-company comparison using modified TOPSIS with objective weights. Computers & Operations Research. 2000;27(10):963–73.

[27] Lin C-L, Shih Y-H, Tzeng G-H, Yu H-C. A service selection model for digital music service platforms using a hybrid MCDM approach. Applied Soft Computing. 2016;48:385–403. 10.1016/j.asoc.2016.05.035

[28] Rao RV. A decision making methodology for material selection using an improved compromise ranking method. Materials & Design. 2008;29(10):1949–54.

[29] Chang T-H. Fuzzy VIKOR method: a case study of the hospital service evaluation in Taiwan. Information Sciences. 2014;271:196–212.

[30] Rao Hill S, Troshani I. Factors influencing the adoption of personalisation mobile services: empirical evidence from young Australians. International Journal of Mobile Communications. 2010;8(2):150–68.

[31] Teng W, Lu H-P, Yu H. Exploring the mass adoption of third-generation (3G) mobile phones in Taiwan. Telecommunications Pol. 2009;33(10):628–41.

[32] Zhou T. Examining mobile banking user adoption from the perspectives of trust and flow experience. Information Technology and Management. 2012;13(1):27–37.

[33] Yoon C, Jeong C, Rolland E. Understanding individual adoption of mobile instant messaging: a multiple perspectives approach. Information Technology and Management. 2015;16(2):139–51. 10.1007/s10799-014-0202-4

[34] Barnes SJ. The mobile commerce value chain: analysis and future developments. International journal of information management. 2002;22(2):91–108.

[35] Blechar J, Constantiou ID, Damsgaard J. Exploring the influence of reference situations and reference pricing on mobile service user behaviour. European Journal of Information Systems. 2006;15(3):285–91.

[36] Bowers CP, Creed C, Cowan BR, Beale R. Touching annotations: A visual metaphor for navigation of annotation in digital documents. Int J Hum Comput Stud. 2013;71(12):1103–11.

[37] Pagani M. Determinants of adoption of third generation mobile multimedia services. Journal of interactive marketing. 2004;18(3):46–59.

[38] Lee PT-W, Lin C-W, Shin S-H. A comparative study on financial positions of shipping companies in Taiwan and Korea using entropy and grey relation analysis. Expert Systems with Applications. 2012;39(5):5649–57. 10.1016/j.eswa.2011.11.052

[39] Zadeh LA. Fuzzy sets as a basis for a theory of possibility. Fuzzy sets and systems. 1978;1(1):3–28.

[40] Quang NH, Yu VF, Lin AC, Dat LQ, Chou S-Y. Parting curve selection and evaluation using an extension of fuzzy MCDM approach. Applied Soft Computing. 2013.

[41] Chou Y-C, Sun C-C, Yen H-Y. Evaluating the criteria for human resource for science and technology (HRST) based on an integrated fuzzy AHP and fuzzy DEMATEL approach. Applied Soft Computing. 2011.

[42] Mokhtarian M, Hadi-Vencheh A. A new fuzzy TOPSIS method based on left and right scores: An application for determining an industrial zone for dairy products factory. Applied Soft Computing. 2012 10.1016/j.asoc.2011.12.003

[43] Kang D, Jang W, Park Y. Evaluation of e-commerce websites using fuzzy hierarchical TOPSIS based on E-S-QUAL. Applied Soft Computing. 2016;42:53–65. 10.1016/j.asoc.2016.01.017

[44] Chen S-H, Wang C-C, editors. Properties of fuzzy distance of LR type fuzzy numbers. 2008 International Conference on Machine Learning and Cybernetics; 2008.

[45] Chen VYC, Lien H-P, Liu C-H, Liou JJH, Tzeng G-H, Yang L-S. Fuzzy MCDM approach for selecting the best environment-watershed plan. Applied Soft Computing. 2011;11(1):265–75. 10.1016/j.asoc.2009.11.017

[46] Efe B. An integrated fuzzy multi criteria group decision making approach for ERP system selection. Applied Soft Computing. 2016;38:106–17.

[47] Dong M, Li S, Zhang H. Approaches to group decision making with incomplete information based on power geometric operators and triangular fuzzy AHP. Expert Systems with Applications. 2015;42(21):7846–57.

[48] Zhu B, Xu Z. Analytic hierarchy process-hesitant group decision making. European Journal of Operational Research. 2014;239(3):794–801.

[49] Liu S, Chan FTS, Ran W. Decision making for the selection of cloud vendor: An improved approach under group decision-making with integrated weights and objective/subjective attributes. Expert Systems with Applications. 2016;55:37–47. 10.1016/j.eswa.2016.01.059

[50] Fu C, Wang Y. An interval difference based evidential reasoning approach with unknown attribute weights and utilities of assessment grades. Computers & Industrial Engineering. 2015;81:109–17.

[51] Chin K-S, Fu C, Wang Y. A method of determining attribute weights in evidential reasoning approach based on incompatibility among attributes. Computers & Industrial Engineering. 2015;87:150–62.

[52] Wan S-P, Xu G-l, Wang F, Dong J-y. A new method for Atanassov’s interval-valued intuitionistic fuzzy MAGDM with incomplete attribute weight information. Information Sciences. 2015;316:329–47.

[53] Cui L, Chan HK, Zhou Y, Dai J, Lim JJ. Exploring critical factors of green business failure based on Grey-Decision Making Trial and Evaluation Laboratory (DEMATEL). J Bus Res. 2019;98:450–61. 10.1016/j.jbusres.2018.03.031

[54] Acuña-Carvajal F, Pinto-Tarazona L, López-Ospina H, Barros-Castro R, Quezada L, Palacio K. An integrated method to plan, structure and validate a business strategy using fuzzy DEMATEL and the balanced scorecard. Expert Systems with Applications. 2019;122:351–68. 10.1016/j.eswa.2019.01.030

[55] Asan U, Kadaifci C, Bozdag E, Soyer A, Serdarasan S. A new approach to DEMATEL based on interval-valued hesitant fuzzy sets. Applied Soft Computing. 2018;66:34–49. 10.1016/j.asoc.2018.01.018

[56] Quezada LE, López-Ospina HA, Palominos PI, Oddershede AM. Identifying causal relationships in strategy maps using ANP and DEMATEL. Computers & Industrial Engineering. 2018;118:170–9. 10.1016/j.cie.2018.02.020

[57] Tzeng G-H, Chiang C-H, Li C-W. Evaluating intertwined effects in e-learning programs: A novel hybrid MCDM model based on factor analysis and DEMATEL. Expert systems with Applications. 2007;32(4):1028–44.

[58] Baykasoğlu A, Gölcük İ. Development of an interval type-2 fuzzy sets based hierarchical MADM model by combining DEMATEL and TOPSIS. Expert Systems with Applications. 2017;70:37–51. 10.1016/j.eswa.2016.11.001

[59] Shahraki AR, Paghaleh MJ. Ranking the voice of customer with fuzzy DEMATEL and fuzzy AHP. Indian Journal of Science and Technology. 2011;4(12):1763–72.

[60] Shannon C-W, Weaver W. W (1949) The mathematical Theory of Communication. Press UoI, editor. 1948.

[61] Zitnick CL, Kanade T, editors. Maximum entropy for collaborative filtering. Proceedings of the 20th conference on Uncertainty in artificial intelligence; 2004: AUAI Press.

[62] Wu S, Fu Y, Shen H, Liu F. Using ranked weights and Shannon entropy to modify regional sustainable society index. Sustainable Cities and Society. 2018;41:443–8. 10.1016/j.scs.2018.05.052

[63] Song M, Zhu Q, Peng J, Santibanez Gonzalez EDR. Improving the evaluation of cross efficiencies: A method based on Shannon entropy weight. Computers & Industrial Engineering. 2017;112:99–106. 10.1016/j.cie.2017.07.023

[64] Li X, Wang K, Liu L, Xin J, Yang H, Gao C. Application of the entropy weight and TOPSIS method in safety evaluation of coal mines. Procedia Engineering. 2011;26:2085–91.

[65] Opricovic S, Tzeng G-H. Compromise solution by MCDM methods: A comparative analysis of VIKOR and TOPSIS. European journal of operational research. 2004;156(2):445–55.

[66] Dincer H, Hacioglu U. Performance evaluation with fuzzy VIKOR and AHP method based on customer satisfaction in Turkish banking sector. Kyb. 2013;42(7):1072–85.

[67] Kim Y, Chung E-S. Fuzzy VIKOR approach for assessing the vulnerability of the water supply to climate change and variability in South Korea. Appl Math Model. 2013;37(22):9419–30.

[68] Opricovic S. Multicriteria optimization of civil engineering systems. Faculty of Civil Engineering, Belgrade. 1998;2(1):5–21.

[69] Büyüközkan G, Ruan D. Evaluation of software development projects using a fuzzy multi-criteria decision approach. Mathematics and Computers in Simulation. 2008;77(5–6):464–75. 10.1016/j.matcom.2007.11.015

[70] Gul M, Ak MF, Guneri AF. Pythagorean fuzzy VIKOR-based approach for safety risk assessment in mine industry. J Saf Res. 2019;69:135–53. 10.1016/j.jsr.2019.03.005 31235225

[71] Zeng S, Chen S-M, Kuo L-W. Multiattribute decision making based on novel score function of intuitionistic fuzzy values and modified VIKOR method. Information Sciences. 2019;488:76–92. 10.1016/j.ins.2019.03.018

[72] Ren Z, Xu Z, Wang H. Dual hesitant fuzzy VIKOR method for multi-criteria group decision making based on fuzzy measure and new comparison method. Information Sciences. 2017;388–389:1–16. 10.1016/j.ins.2017.01.024

[73] Zhang H, Peng Y, Hou L, Tian G, Li Z. A hybrid multi-objective optimization approach for energy-absorbing structures in train collisions. Information Sciences. 2019;481:491–506.

[74] Tian G, Zhang H, Feng Y, Jia H, Zhang C, Jiang Z, et al Operation patterns analysis of automotive components remanufacturing industry development in China. Journal of cleaner production. 2017;164:1363–75.

[75] Ploskas N, Papathanasiou J. A decision support system for multiple criteria alternative ranking using TOPSIS and VIKOR in fuzzy and nonfuzzy environments. Fuzzy Sets and Systems. 2019 10.1016/j.fss.2019.01.012

[76] Tian G, Liu X, Zhang M, Yang Y, Zhang H, Lin Y, et al Selection of take-back pattern of vehicle reverse logistics in China via Grey-DEMATEL and Fuzzy-VIKOR combined method. Journal of Cleaner Production. 2019;220:1088–100.

[77] Chincholle D, Goldstein M, Nyberg M, Eriksson M, editors. Lost or found? A usability evaluation of a mobile navigation and location-based service. International Conference on Mobile Human-Computer Interaction; 2002: Springer.

[78] Chen C, Liao G-P, Shi X-H, Zhao X. The Model of User Interest Update and User Classification in Personal Information Push Service. Procedia Environmental Sciences. 2011;10:262–8.

[79] Dijkstra J, de Vries B, Jessurun J. Wayfinding search strategies and matching familiarity in the built environment through virtual navigation. Transportation Research Procedia. 2014;2:141–8.

[80] Moreno M, Shahrabadi S, José J, du Buf JH, Rodrigues JM. Realtime local navigation for the blind: detection of lateral doors and sound interface. Procedia Computer Science. 2012;14:74–82.

[81] Itoh N, Yamashita A, Kawakami M, editors. Effects of car-navigation display positioning on older drivers' visual search. Int Congr Ser; 2005: Elsevier. 10.1016/j.ics.2005.07.090

[82] Chong J-L, Chong AY-L, Ooi K-B, Lin B. An empirical analysis of the adoption of m-learning in Malaysia. International Journal of Mobile Communications. 2011;9(1):1–18.

[83] Browne K, Anand C. An empirical evaluation of user interfaces for a mobile video game. Entertainment Computing. 2012;3(1):1–10.

[84] Alnanih R, Ormandjieva O, Radhakrishnan T. Context-based and rule-based adaptation of mobile user interfaces in mHealth. Procedia Computer Science. 2013;21:390–7.

