# Correction: Evaluating mobile services using integrated weighting approach and fuzzy VIKOR

**DOI:** 10.1371/journal.pone.0222312

**Published:** 2019-09-05

**Authors:** Yongyoon Suh, Yongtae Park, Daekook Kang

There are errors in the Funding statement. The correct Funding statement is as follows: This work was supported by the National Research Foundation (NRF) of Korea Grant funded by the Korean Government (MSIT) (2019R1G1A1006073).
